# A tailored MoS_2_ membrane with strong DNA-binding capability enhances aquatic biota detection through environmental DNA metabarcoding

**DOI:** 10.1093/nsr/nwag055

**Published:** 2026-01-29

**Authors:** Liang Mei, Chun Ming How, Mingzi Sun, Ruixin Yan, Weikang Zheng, Yue Zhang, Honglu Hu, Bolong Huang, Jian-Wen Qiu, Zhiyuan Zeng, Kenneth M Y Leung

**Affiliations:** Department of Materials Science and Engineering, City University of Hong Kong, Hong Kong 999077, China; State Key Laboratory of Marine Environmental Health, City University of Hong Kong, Hong Kong 999077, China; School of Chemistry and Chemical Engineering, South China University of Technology, Guangzhou 510641, China; State Key Laboratory of Marine Environmental Health, City University of Hong Kong, Hong Kong 999077, China; Department of Biology, Hong Kong Baptist University, Hong Kong 999077, China; National Observation and Research Station of Coastal Ecological Environments in Macao, Macau Environmental Research Institute, Macau University of Science and Technology, Macau 999078, China; Department of Chemistry, City University of Hong Kong, Hong Kong 999077, China; Department of Materials Science and Engineering, City University of Hong Kong, Hong Kong 999077, China; Department of Materials Science and Engineering, City University of Hong Kong, Hong Kong 999077, China; Department of Materials Science and Engineering, City University of Hong Kong, Hong Kong 999077, China; Department of Materials Science and Engineering, City University of Hong Kong, Hong Kong 999077, China; Department of Chemistry, City University of Hong Kong, Hong Kong 999077, China; State Key Laboratory of Marine Environmental Health, City University of Hong Kong, Hong Kong 999077, China; Department of Biology, Hong Kong Baptist University, Hong Kong 999077, China; Department of Materials Science and Engineering, City University of Hong Kong, Hong Kong 999077, China; State Key Laboratory of Marine Environmental Health, City University of Hong Kong, Hong Kong 999077, China; Shenzhen Research Institute, City University of Hong Kong, Shenzhen 518057, China; State Key Laboratory of Marine Environmental Health, City University of Hong Kong, Hong Kong 999077, China; Department of Chemistry, City University of Hong Kong, Hong Kong 999077, China; Shenzhen Research Institute, City University of Hong Kong, Shenzhen 518057, China

**Keywords:** MoS_2_ membrane, environmental DNA, S–N/O bond, capture efficiency, biodiversity detection

## Abstract

Active environmental DNA (eDNA) sampling, typically involving water filtration, offers the advantage of capturing relatively high concentrations of eDNA, making it particularly valuable in aquatic environments with low DNA concentrations. However, this approach faces several limitations, including low DNA-capture efficiency, limited molecular selectivity and the risk of contamination during filtration and handling. Moreover, commonly used membrane materials often lack strong and specific DNA-binding affinity, which reduces detection sensitivity and compromises biodiversity assessment. To address these challenges, we developed a MoS_2_-coated mixed cellulose ester membrane that significantly enhances both the efficiency and the selectivity in capturing eDNA. The MoS_2_ coating facilitates preferential interactions with DNA via van der Waals forces between the sulfur atoms in MoS_2_ and the oxygen or nitrogen atoms in DNA bases. Laboratory and field tests with marine fish confirmed that the MoS_2_-coated membrane significantly enhanced the selectivity and sensitivity of eDNA-based biota detection. This work represents the first application of a 2D nanomaterial-based membrane for eDNA collection and detection. Furthermore, the membrane is low-cost and scalable, and requires no further processing. This invention offers a promising strategy for the development of 2D nanomaterials-based eDNA-sampling tools and showcases their broader potential in environmental biotechnology applications.

## Teaser

This work presents a MoS_2_ membrane with abundant active sulfur sites enabling efficient environmental DNA capture and accurate aquatic biodiversity detection, opening a new avenue for 2D materials in biological applications.

## INTRODUCTION

Monitoring marine biodiversity is essential for understanding ecosystem health, guiding conservation efforts and informing fisheries management [1]. In recent years, environmental DNA (eDNA) has emerged as a noninvasive and highly sensitive approach for detecting aquatic organisms, enabling the identification of species from trace genetic material shed into water, soil and air [2]. Among various ecosystems, marine environments pose particular challenges for eDNA analysis due to the rapid dilution, degradation and dispersion of genetic material [3]. Active eDNA sampling, which typically involves filtering large volumes of water through porous membranes, remains the most widely used method due to its high efficiency and control over sampling parameters. However, current membrane materials suffer from several limitations, including low DNA-capture efficiency, limited molecular selectivity and the risk of introducing background contamination during handling and filtration [4]. These issues are especially critical in marine systems, in which the low abundance of target DNA requires more efficient and selective capture platforms [5–7].

Two-dimensional materials have emerged as promising platforms for biomolecular detection due to their large surface area, unique electronic properties and capacity for direct interaction with nucleic acids [8,9]. Among them, transition metal dichalcogenides have been widely explored for DNA sensing. A series of experimental studies have shown that MoS_2_ [10] and related 2D chalcogenides (TaS_2_, TiS_2_, Ta_2_NiS_5_) [11,12] can serve as universal fluorescence quenchers with selective adsorption toward DNA. This selectivity arises from van der Waals interactions between nucleobases and the 2D nanosheet surface, allowing DNA to be strongly adsorbed and quenched.

In addition to these experimental efforts, theoretical studies have also validated the potential of MoS_2_ for DNA detection. First-principles simulations revealed that individual DNA bases exhibit distinct binding energies and electronic signatures when adsorbed on MoS_2_ surfaces, enabling base-level discrimination [13]. Moreover, a recent comparative molecular dynamics study systematically analysed DNA translocation through nanopores in 2D materials (graphene, MoS_2_ and MXene) and highlighted the superior ability of MoS_2_ to differentiate between base types based on ionic current and interaction dynamics [14].

Together, these experimental and computational studies provide a strong empirical and theoretical foundation for utilizing MoS_2_ in DNA detection. However, its application in eDNA sampling, particularly under field conditions such as in marine environments, remains largely unexplored. Harnessing these well-established molecular interaction mechanisms in environmental monitoring may unlock new capabilities for high-sensitivity, selective eDNA capture.

**Figure 1.**
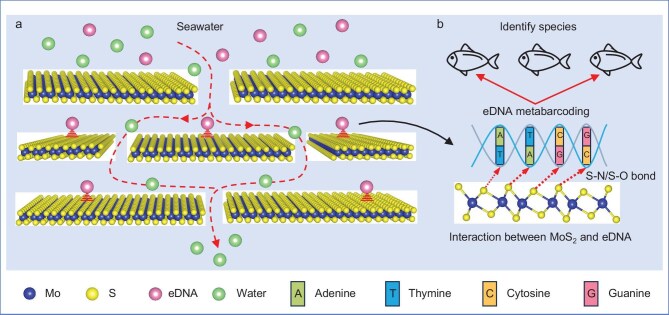
MoS_2_ membrane for aquatic biota detection. (a) Active filtration using a MoS_2_ membrane for eDNA collection. (b) Marine fish detection via eDNA metabarcoding.

Building on these insights, we developed a MoS_2_-coated mixed cellulose ester (MCE) membrane for sampling the eDNA of aquatic biota via membrane filtration (Fig. 1a), with empirical metabarcoding tests using fish-specific primers. This membrane is designed to enhance both sensitivity and selectivity of eDNA capture via preferential base interactions. MoS_2_ contributes superior DNA-binding capabilities by offering abundant adsorption sites and facilitating selective molecular interactions (Fig. 1b), making the membrane highly effective for concentrating trace eDNA from large volumes of seawater. This concentration effect amplifies the target signals, enabling more accurate species identification with a smaller volume of water sample. We demonstrate its performance through laboratory and field metabarcoding assays. This work establishes a foundation for advancing eDNA-sampling technologies by using functional 2D nanomaterials, providing a powerful tool for monitoring marine biodiversity under challenging environmental conditions. Ultimately, this study contributes to the growing field of molecular ecology by bridging nanotechnology and environmental genomics to enable more sensitive, efficient approaches to biodiversity monitoring.

## RESULTS AND DISCUSSION

### Single-layer MoS**_2_** preparation and characterization

**Figure 2.**
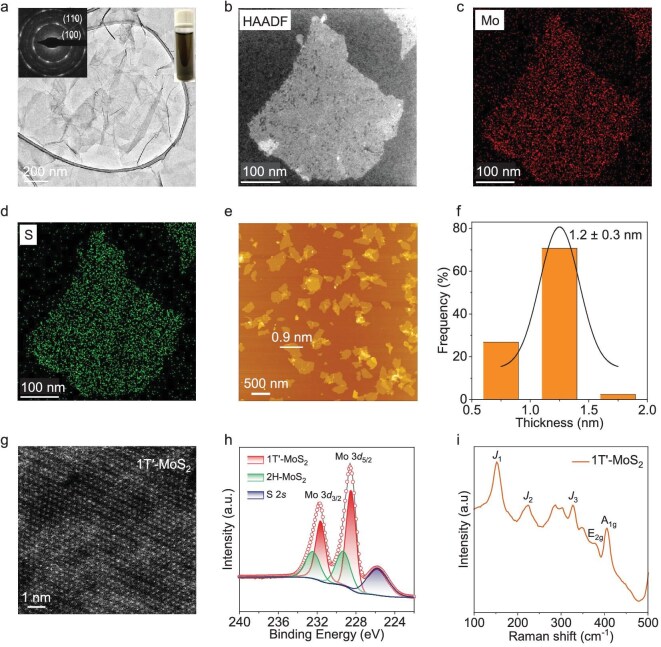
MoS_2_ nanosheet preparation and characterization. (a) TEM image of MoS_2_ NSs. Insets show the diffraction pattern and photograph of exfoliated MoS_2_ NSs solution. (b–d) Corresponding elemental mapping (Mo and S). (e) AFM image of exfoliated MoS_2_ NSs. (f) Thickness-distribution histogram of MoS_2_ NSs measured by AFM (1.2 nm is the mean thickness and 0.3 nm is the SD). (g) Annular dark-field scanning transmission electron microscopy image of typical 1T′-MoS_2_ NSs. (h) X-ray photoelectron spectroscopy and (i) Raman spectra of exfoliated 1T′-MoS_2_ NSs. AFM, atomic force microscopy.

The 2D MoS_2_ nanosheets (NSs) were synthesized via an electrochemical lithium-ion (Li^+^) intercalation-assisted exfoliation method (see Fig. S1 and the ‘Methods’ section in the online Supplementary file for details) [15–17]. In this process, the Li^+^ ions were intercalated into the van der Waals gaps of bulk MoS_2_ (Figs S2 and S3) under an electrochemical driving force, followed by sonication and exfoliation in deionized (DI) water. Due to their negatively charged surfaces—confirmed by the zeta potential of −60 mV (Fig. S4)—the exfoliated MoS_2_ NSs are well dispersed in the DI water (inset in Fig. 2a).

Figure 2a shows the transmission electron microscopy (TEM) images of typical exfoliated MoS_2_ NSs, which exhibit lateral sizes ranging from 200 to 500 nm. The selected area electron diffraction pattern displays two sets of 6-fold symmetric diffraction spots corresponding to the (100) and (110) crystallographic planes of MoS_2_ [18]. Energy-dispersive X-ray spectroscopy (EDS) mapping (Fig. 2b–d) confirms the uniform distribution of molybdenum (Mo) and sulfur (S) across all of the nanosheets. Atomic force microscopy (AFM) analysis reveals a thickness of 1.2 ± 0.3 nm for the exfoliated NSs (Fig. 2e and f), indicating that the majority are monolayers [19]. The structure of the exfoliated MoS_2_ NSs was confirmed by using aberration-corrected annular dark-field scanning transmission electron microscopy (ADF-STEM). The ADF-STEM image reveals zigzag atomic chains with a minimum Mo–Mo distance of 2.77 Å (Fig. 2g), consistently with the theoretical 1T′ structure [20]. X-ray photoelectron spectroscopy (XPS) shows a dominant Mo 3*d* doublet at 228.5 and 231.6 eV (Mo 3*d*_5/2_ and Mo 3*d*_3/2_, respectively; Fig. 2h), further supporting the 1T′ phase [21]. Additionally, the Raman spectrum exhibits five characteristic peaks at 151.4 (*J*_1_), 223.7 (*J*_2_), 327.6 (*J*_3_), 379.1 (E_2g_) and 404.9 cm^−1^ (A_1g_) (Fig. 2i), confirming the dominant 1T′ structure [21], in agreement with the ADF-STEM and XPS analyses. Accurate determination of the MoS_2_ structure is essential for correlating structural features with performance in subsequent theoretical calculations.

### MoS**_2_** membrane preparation and characterization

**Figure 3.**
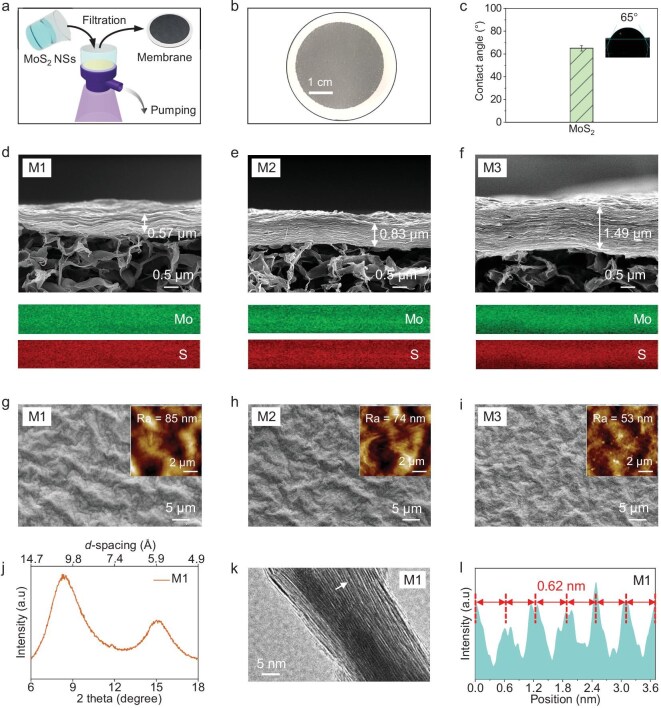
MoS_2_ membrane preparation and characterization. (a) Schematic of MoS_2_ membrane preparation. (b) Photograph and (c) contact angle of MoS_2_ membrane (M1). Scanning electron microscopy images of the (d−f) membrane surface (insets show the AFM images of the membrane surface, where Ra is the average roughness; below the images are the corresponding elemental mapping analyses) and (g−i) cross section. (j) X-ray diffraction pattern of a wet M1 membrane. (k) TEM cross-sectional image of a dried M1 membrane. (l) Corresponding intensity profile along the white arrow in (k) shows the interlayer spacing of the dried M1 membrane.

The MoS_2_ membrane was prepared by the vacuum filtration of exfoliated nanosheets onto MCE substrate (see Fig. 3a and b and the Methods section in the online Supplementary file for details) [22]. The membrane thickness was tuned by controlling the mass of the filtered MoS_2_ NSs. A series of membranes with varying thicknesses—designated as M1, M2 and M3—were fabricated accordingly, while M0 refers to the pristine MCE substrate. The water affinity of the membrane was assessed by using contact-angle measurements, showing a value of 65° (Fig. 3c), indicating its hydrophilic nature and suitability for aqueous-based applications. Cross-sectional scanning electron microscopy (SEM) images revealed the laminated structures of M1, M2 and M3 (Fig. 3d–f), with corresponding thicknesses of 0.57, 0.83 and 1.49 μm, respectively. EDS mapping confirmed the homogeneous distribution of Mo and S elements across the membranes.

Top-view SEM (Fig. 3g–i and Fig. S5) and AFM images (insets in Fig. 3g–i and Fig. S6) indicated that the membrane surface became progressively smoother with increasing thickness. The average surface roughness (Ra) values decreased from 85 nm for M1 to 74 nm for M2 and further to 53 nm for M3. X-ray diffraction (XRD) patterns of the wet MoS_2_ membrane showed a dominant peak at 8.3° (Fig. 3j), corresponding to an interlayer spacing of 10.6 Å. Subtracting the 6.2 Å thickness of a single MoS_2_ layer (including the van der Waals radii of two sulfur atoms) yields a capillary width of 4.4 Å, which is sufficient to allow the passage of water molecules. However, cross-sectional TEM images revealed an interlayer spacing of 6.2 Å after drying (Fig. 3k and l), with a nearly zero capillary width, which is too narrow for water molecules to permeate. Therefore, to preserve the permeability of the membrane, it should be maintained in a hydrated (wet) state prior to filtration experiments.

Hence, the developed MoS_2_ membrane features a laminated structure with a hydrophilic surface and an interlayer spacing of ∼1 nm, enabling effective filtration and separation [23]. The interlayers consist of single-layer MoS_2_ NSs with high surface area and activity, which can adsorb molecules during permeation [24]. By harnessing the unique properties of the MoS_2_ membrane—including strong adsorptivity [10], molecular sieving capabilities [25], high permeability [26] and excellent chemical stability [27]—we anticipate significant advantages in eDNA sampling: (i) enhanced eDNA capture efficiency—the unique DNA-adsorption properties of the MoS_2_ membrane, combined with their laminar structures, enable the effective capture and retention of eDNA fragments, thereby improving sensitivity and detection rates; (ii) reduced contamination risk—the MoS_2_ membrane can function as a physical barrier with tunable interlayer spacing, helping to block the passage of larger contaminants and minimize background noise; and (iii) simplified sampling workflow—the integration of a MoS_2_ membrane streamlines the eDNA-sampling process by eliminating additional sample-processing steps, reducing both time and cost while minimizing potential handling errors. These advantages make the MoS_2_ membrane a promising platform for advancing high-sensitivity eDNA-sampling technologies.

### Laboratory testing of MoS**_2_** membrane for marine fish eDNA detection

**Figure 4.**
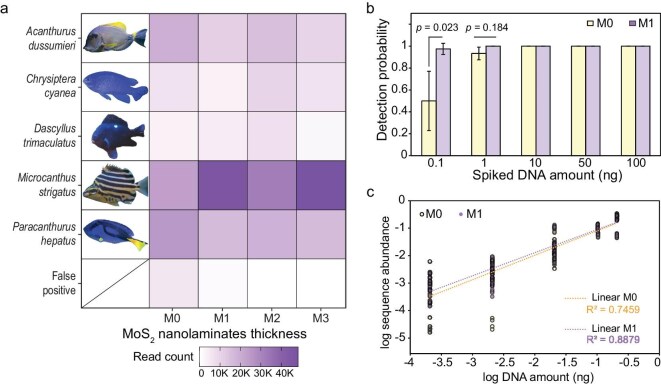
Detection of marine fish under laboratory conditions. (a) Aquarium water (mixed from four tanks, 1 L per tank, totaling 4 L) was collected and filtered through MCE membranes coated with varying thicknesses of MoS_2_ (denoted as M0, M1, M2 and M3), followed by eDNA extraction, PCR amplification and high-throughput sequencing. After the data were processed, the results were rarefied to the exact total reads for Class Actinopteri (ray-finned fish). Any fish identified as absent from the laboratory aquarium was considered a false positive. The authors captured images of the fish directly from the aquarium (see Fig. S7 for details). (b) Tissue DNA from various marine fish species was mixed in varying amounts to form a mock community (see Table S3). The mock-community DNA was spiked into sterilized MilliQ water, mixed thoroughly and filtered onto M0 and M1 membranes at a flow rate of 0.002 L min^−1^. The samples were processed by using the same methodologies. The non-target reads were filtered and the presence/absence of the fish taxa in each sample was determined. The detection probability of the taxa was calculated by dividing the number of occurrences by the total sample size (*n* = 10). The detection probability data for different taxa were then grouped by input DNA amount. (c) Linear regressions of the log-transformed relative sequence abundance detected in the mock-community assays against the log-transformed relative DNA amount.

Previous studies have reported that low filtration volumes may substantially underestimate biodiversity, likely due to insufficient sampling effort leading to unrepresentative results [28]. Filtration volumes of 2–4 L are generally recommended. In our aquarium trials, 1 L of artificial seawater was pooled from each of four tanks (4 L in total) containing five coral reef fish species (i.e. *Acanthurus dussumieri, Chrysiptera cyanea, Dascyllus trimaculatus, Microcanthus strigatus* and *Paracanthurus hepatus*, see Fig. S7). The water was filtered through MCE membranes coated with varying thicknesses of MoS_2_ NSs (M0, M1, M2 and M3). DNA was immediately extracted from the membranes after filtration to assess the eDNA-metabarcoding efficiency (see Fig. S8 and Methods section in the online Supplementary file for details). The aquarium sequencing runs yielded an average of 2.1 ± 0.9 million reads per sample, with >96% of reads retained after quality filtering, merging and the removal of chimeric sequences (Table S1). Rarefaction curves approached asymptotes for all samples (M0–M3, Fig. S9a), indicating sufficient sequencing depth. Following taxonomic assignment, taxa filtering and rarefaction, 29 fish taxa were identified at the genus and species levels. After the removal of taxa with reads of <10 and occurring in fewer than two samples, eight unique taxa were detected in total and the taxa that were not present in the tank were considered false positives. The occurrence of false positives may be attributed to the open lids of the aquariums, the use of natural feed (i.e. mussels), the introduction of captured invertebrate specimens (hence the natural seawater) into the tank after our fieldwork and PCR contamination. These false positives also highlight the importance of careful sample handling when conducting an eDNA study in a closed system and demonstrate the sensitivity of eDNA metabarcoding to trace amounts of DNA in the water column. The target species comprises 93.4%–99.9% of the total informative reads (Table S2). All coated membranes (M1, M2 and M3) successfully detected the target aquarium fish, while the uncoated membrane (M0) exhibited the highest proportion of non-target fish reads (Fig. 4a). The target-read abundance varied across replicates, with *M. strigatus* (Stripey) and *P. hepatus* (Blue Tang) consistently showing the highest read counts.

To further investigate whether the MoS_2_ nanolaminate coating enhances the sensitivity of the eDNA-metabarcoding assay, we conducted a mock-community assay by mixing various fish DNA samples and filtering them by using the M0 and M1 membranes. The DNA was then immediately extracted and processed. An average of 1.3 ± 0.99 million reads per sample was generated, with >90% of reads retained after quality filtering, merging and chimaera removal (Table S1). The sequencing depth was considered sufficient, as indicated by rarefaction curves approaching asymptotes for all samples (Fig. S9b). Overall, all fish species were detected in both membranes, with the target fish sequence reads comprising >84% of the fish reads (Tables S3 and S4). Again, the presence of trace amounts of non-target reads might be attributable to the eDNA residues in the specimens that were co-extracted and amplified. The results showed that the M1 membranes exhibited markedly improved detection probability for low-abundance DNA (0.1 ng) compared with M0 membranes (0.98 vs. 0.50, *P* = 0.023) (Fig. 4b and Table S3). There was no difference in the detection probability of high-abundance DNA (1–100 ng) between M0 and M1 (Fig. 4b and Table S3). In addition, the M1 membranes exhibited markedly improved detection of *Echeneis naucrates*, which was only detected once out of 10 replicates in the M0 samples (Tables S3 and S4). Notably, the log-transformed sequence abundance correlated well with the log-transformed relative DNA amount of fish taxa and the linear correlation was slightly better in the M1 (*R*^2^ = 0.8879) than in the M0 (*R*^2^ = 0.7459) samples (Fig. 4c). Additionally, to determine the adsorption kinetics and maximum amount of eDNA that the MoS_2_ membrane can collect, additional adsorption assays were conducted (see Methods section in the online Supplementary file for details). The results show that, under laboratory conditions, with an input DNA concentration of 22 ng µL^−1^, the times to saturation were 0.98 min for the M0 and 2.24 min for the M1 membranes (Fig. S10). We also find that the MoS_2_ nanolaminate coating substantially improves the adsorption of free DNA, increasing the maximum experimental adsorption capacity (*q*_e_) by 128% (from 0.88 to 2.01 mg g^−1^) and the maximum theoretical adsorption capacity (*q*_0_) by 131% (from 0.54 to 1.25 mg g^−1^), potentially contributing to the enhanced sensitivity of the eDNA-metabarcoding assays. These results confirm that the MoS_2_-coating procedure enhances fish detection using eDNA metabarcoding, possibly by improving free eDNA adsorption, supporting the suitability of the modified membranes for field applications.

### Field testing of MoS**_2_** membrane for marine fish eDNA detection

**Figure 5.**
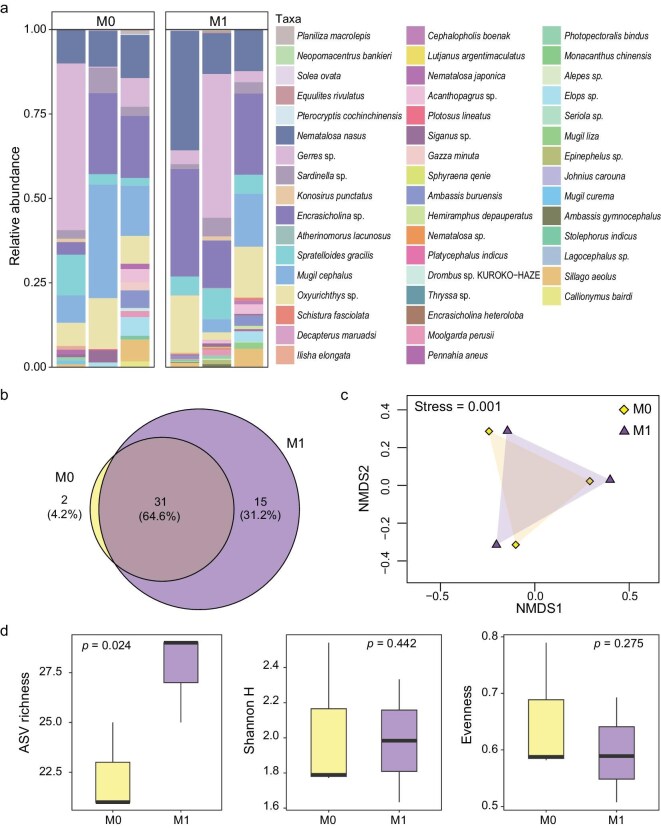
Field testing of MoS_2_ membranes for marine fish eDNA detection. Water samples were collected from a random site at the Hoi Ha Wan Marine Park, Hong Kong, with a sampling effort of three replicates × 4 L per condition. Only sequences from ray-finned fish were retained for downstream analysis. (a) Overview of the fish taxa detected in the pristine MCE membrane and the coated M1 membrane. (b) Venn diagram showing the overlapping and exclusive taxa detected in each membrane. (c) NMDS ordination of the samples based on their Bray–Curtis dissimilarities. (d) Biodiversity metrics, including amplicon sequence variants of richness, Shannon H and evenness, were calculated from the fish detected by each membrane. Pairwise comparisons were conducted by using Student’s *t*-tests.

To further evaluate whether the MoS_2_ NSs coating enhances eDNA-based marine fish detection under field conditions, water samples were collected from the Hoi Ha Wan Marine Park, Hong Kong (Fig. S11) and filtered *in situ* using MoS_2_-coated membranes (see Methods section in the online Supplementary file for details). MoS_2_ membranes of varying thickness (M1, M2 and M3) were fabricated to assess their impact on the filtration efficiency and eDNA collection. Results indicate that increasing the thickness markedly decreases the filtration speed (Fig. S12). The DNA yield initially increases but then decreases with thickness (M2 > M3 > M1, Fig. S13), while the fish-detection sensitivity remains largely unchanged (Fig. 4a), likely because longer filtration times increase the risk of sample contamination. Accordingly, only the M1 membrane was selected for comparison with the uncoated M0 membrane. After eDNA collection, XPS spectra revealed a strong P 2*p* signal on the M1 membrane (Fig. S14), whereas no such signal was observed on M1 prior to filtration (Fig. S15) or on the uncoated M0 membrane (Fig. S16). These results confirm that the MoS_2_-coated M1 membrane effectively captures eDNA during filtration. To ensure that the MoS_2_ coating does not significantly affect DNA extraction, we further evaluated the quantity and quality of DNA extracted from the M0 and M1 membranes (Fig. S17). The results show that both membranes yield similar DNA yields, purity (A260/280), ssDNA/dsDNA ratio and GC content, while they differed slightly in the fragment-length distribution (Fig. S17a–e). The fragments with lengths of between 100 and 15 000 bp indicate the presence of different states of eDNA in marine water, including tissue DNA, free DNA and fragmented DNA (Fig. S17e). After PCR amplification and sequencing, an average of 1.6 ± 0.3 million reads per sample was generated, with >97% of reads retained after quality filtering, merging and chimaera removal (Table S1). The sequencing depth was considered sufficient, as indicated by rarefaction curves approaching asymptotes for all samples (Fig. S9c). After taxonomic assignment, taxa filtering and rarefaction, a total of 48 fish taxa were identified (Table S5). The dominant taxa detected at the sampling site included Bloch’s gizzard shad (*Nematalosa nasus*), anchovies (*Gerres* sp. and *Encrasicholina* sp.), silver-stripe round herring (*Spratelloides gracilis*), flathead gray mullet (*Mugil cephalus*) and arrow-fin goby (*Oxyurichthys* sp.) (Fig. 5a). Among the identified taxa, 31 were shared between the two membrane types, while the uncoated M0 exclusively detected 2, and 15 were uniquely detected by the coated M1 (Fig. 5b). Based on Bray–Curtis dissimilarities, non-metric multidimensional scaling (NMDS) analysis revealed a substantial overlap in fish communities detected by both membrane types, with a low stress value (0.001) indicating a good fit (Fig. 5c). Although the M1 membrane yielded significantly higher amplicon sequence variants richness than M0 (27.7 vs. 22.3, *P* = 0.024), no significant differences were observed in Shannon diversity or evenness indices (*P* > 0.05) (Fig. 5d). These results suggested that the MoS_2_-coated membrane enhanced the detection sensitivity without altering the overall community composition in the field test.

To investigate whether the eDNA adsorption occurs only on the membrane surface or also within interlayer galleries, cross-sectional SEM–energy dispersive X-ray spectroscopy (EDX) mapping and XPS etch analyses were performed. As shown in Figs S18 and S19, both the SEM–EDX and the XPS results indicate that eDNA is adsorbed on both the membrane surface and within the interlayer galleries. Furthermore, after eDNA sampling, the structure, morphology and composition of the M1 membrane remain stable, as confirmed by XRD (Fig. S20), cross-sectional SEM imaging (Fig. S21), XPS analysis (Fig. S14c) and Raman spectroscopy (Fig. S22). In particular, Mo leaching into the permeate was carefully evaluated and found to be well below the WHO guideline value (Fig. S23), confirming its ecological safety. The membrane also demonstrates good phase stability and antioxidation performance under practical seawater filtration and air-exposure conditions (Fig. S24). In addition, the M1 membrane shows strong potential for scalable and cost-effective fabrication (Table S6) through gram-scale MoS_2_ nanosheet synthesis (Fig. S25) and the upscaling of the vacuum-filtration process. In the future, the extraction methodologies should be refined, as we found that they may affect the performance of MoS_2_ membranes, with the PowerSoil kit showing better DNA yields than the cetyltrimethylammonium bromide (CTAB)–phenol–chloroform method (Fig. S13).

### Theoretical calculations for mechanism clarification

**Figure 6.**
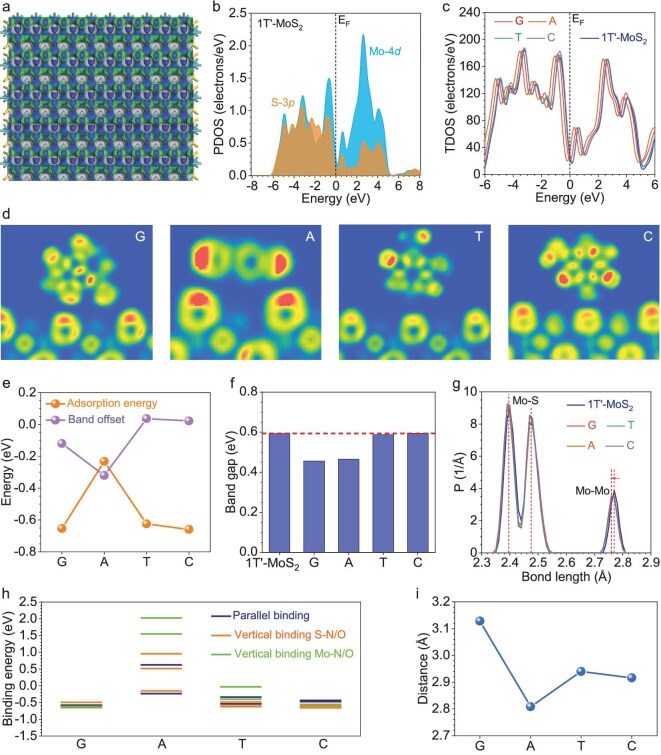
Density functional theory (DFT) calculation for mechanism clarification. (a) Electronic distributions of bonding and antibonding orbitals near the Fermi level in 1T′-MoS_2_. (b) PDOS of 1T′-MoS_2_. (c) TDOS evolutions of 1T′-MoS_2_ adsorbed with different DNA bases. (d) Electron local function of 1T′-MoS_2_ with G, A, T and C bases on the surface. (e) Band offset of TDOS and the binding energies of G, A, T and C bases on 1T′-MoS_2_. (f) Bandgap changes induced by G, A, T and C bases on 1T′-MoS_2_. (g) Bond-length distributions of 1T′-MoS_2_ with G, A, T and C bases. (h) Comparisons of different binding configurations of G, A, T and C bases. (i) Distances between the G, A, T and C bases and the 1T′-MoS_2_ surfaces after relaxation. PDOS, partial density of states; TDOS, total density of states.

To further investigate the interactions between 1T′-MoS_2_ and DNA bases, we selected the fundamental G, A, T and C bases to explore the binding behaviors on 1T′-MoS_2_. First of all, we demonstrated the surface electronic structures of 1T′-MoS_2_ (Fig. 6a). Although the distributions of the bonding and antibonding orbitals are highly ordered on both the Mo and the S sites, we notice slightly different distributions for the two types of S sites. The detailed electronic structures are revealed by the projected partial density of states, in which we notice the good overlapping between the Mo-4*d* and S-3*p* orbitals, supporting the electronic distribution results and stable bonding in the structure (Fig. 6b). Based on the most stable bindings of different DNA bases on 1T′-MoS_2_, we compared the total density of states (TDOS) to explore the influence on the electronic structures (Fig. 6c and Fig. S26). Notably, the corresponding TDOS only exhibits evident shifting while the overall patterns have not been changed, suggesting that the strong interactions between the DNA bases and 1T′-MoS_2_ do not involve chemical-bonding formation. To understand the nature of such interactions, the electron localization function has been presented for G, A, T and C base bindings on 1T′-MoS_2_ with the most stable binding configurations (Fig. 6d and Fig. S26). There are very weak electron localizations between the G, A, T and C bases and 1T′-MoS_2_, indicating that there is no direct chemical bonding formed during the interactions. Regardless of the binding configurations (e.g. vertical, parallel), the detections of the DNA bases by 1T′-MoS_2_ are potentially attributed to the van der Waals interactions. The corresponding binding energies of the most stable binding for different DNA bases are also limited in a relatively small range from –0.23 to –0.65 eV, which is similar to the range of van der Waals interactions (Fig. 6e). Compared with other low-dimensional materials (0.93–1.18 eV for graphene and boron nitride), [13,29] the negative and much lower binding energies of 1T′-MoS_2_ indicate its much higher potential in detecting DNA groups. The bindings of different DNA bases induce distinct responses to the electronic structures, where the G and A groups downshift the TDOS, while the C and T groups upshift the TDOS. Accordingly, the bandgap shows that the binding of G and A decreases the band gap by 0.14 and 0.13 eV, respectively, which is potentially induced by the additional ring in bases A and G to provide a higher possibility of interactions with 1T′-MoS_2_ (Fig. 6f). In contrast, the bindings of base C will only slightly open up the band gap by 0.01 eV. The changes in the band gap follow the order of G > A > T > C. The bond-length analysis of the 1T′-MoS_2_ also proves that the overall bond lengths of the Mo–S bondings remain unchanged (Fig. 6g). The Mo–Mo bondings have been slightly affected, with a minor reduction from 2.77 to 2.76 Å, supporting that the binding of DNA does not significantly affect the structure of the 1T′-MoS_2_ membrane. To realize an in-depth study of the binding behaviors of DNA bases, the binding energies of different configurations have been carefully compared (Fig. 6h). The influences of the solvent environments do not affect the overall binding-energy trends based on selective comparisons (Figs S27 and S28). Based on the binding-energy distributions, the G group shows the lowest sensitivity to the binding configurations, while the binding of the A group is highly correlated with the binding configurations. For the G, C and T bases, the vertical binding on 1T′-MoS_2_ is preferred through S–N/O interactions, while the Mo–N/O interactions are highly unstable, leading to significant increases in the binding energies. These results support that 1T′-MoS_2_ detects the DNA groups mainly through the van der Waals interactions between the N/O and S sites. The binding distances of the DNA bases on 1T′-MoS_2_ also confirm the absence of chemical bondings, where the distances are all >2.8 Å, which is much longer than those of typical chemical bonds (Fig. 6i). Therefore, theoretical calculations have revealed that the detection of DNA by 1T′-MoS_2_ originates from the strong physical adsorptions dominated by the van der Waals interactions.

Based on both experimental and theoretical results, several optimization strategies for MoS_2_ membranes are critical for enabling their practical use in high-throughput eDNA capture. First, tuning the interlayer spacing and adjusting the membrane thickness emerge as effective approaches for balancing hydraulic permeability with adsorption capacity. Second, the intrinsic hydrophilicity of the 1T′-phase MoS_2_ membrane is shown to promote efficient water transport. Third, coating MoS_2_ nanosheets onto porous supports (e.g. fibrous substrates) is identified as a promising route to substantially increase flow rates without sacrificing capture efficiency, representing a key direction for future development.

## CONCLUSIONS

In summary, this study demonstrates for the first time that MoS_2_-coated MCE membranes can enhance eDNA metabarcoding, particularly in marine environments. The coating significantly improves the sensitivity of fish detection, which is attributed to the strong van der Waals interactions between the S atoms in 1T′-MoS_2_ and DNA bases. The membranes can be readily synthesized at scale, used directly for eDNA sampling and coated onto fiber substrates [30,31] to develop the next generation of high-throughput water samplers. Overall, this work provides a simple, rapid and cost-effective approach for aquatic biodiversity monitoring and broadens the biological applications of 2D materials.

## Supplementary Material

nwag055_Supplemental_File

## Data Availability

The sequencing datasets are available online at https://doi.org/10.5061/dryad.3bk3j9kx5.
