# Supplementary material for: A tailored MoS2 membrane with strong DNA-binding capability enhances aquatic biota detection through environmental DNA metabarcoding
Source: Natl Sci Rev. 2026 Jan 29;13(6):nwag055. doi: 10.1093/nsr/nwag055 (PMC13001590; doi:10.1093/nsr/nwag055)
Supplement: nwag055_Supplemental_File [file nwag055_supplemental_file.pdf]

Supporting information

for

A Tailored MoS_2 Membrane with Strong DNA Binding Capability Enhances Aquatic Biota Detection through Environmental DNA Metabarcoding

Liang Mei[1,2,3,†], Chun Ming How[2,4,5,†], Mingzi Sun[6,†], Ruixin Yan[1], Weikang Zheng[1], Yue Zhang[1], Honglu Hu[1], Bolong Huang[6,*], Jian-Wen Qiu[2,4,*], Zhiyuan Zeng[1,2,7,*] and Kenneth M. Y. Leung[2,6,7,*]

[1]Department of Materials Science and Engineering, City University of Hong Kong, Hong Kong 999077, China;

[2]State Key Laboratory of Marine Environmental Health, City University of Hong Kong, Hong Kong 999077, China;

[3]School of Chemistry and Chemical Engineering, South China University of Technology, Guangzhou, 510641, China;

[4]Department of Biology, Hong Kong Baptist University, Hong Kong 999077, China;

[5]National Observation and Research Station of Coastal Ecological Environments in Macao, Macau Environmental Research Institute, Macau University of Science and Technology, Macau 999078, China;

[6]Department of Chemistry, City University of Hong Kong, Hong Kong 999077, China;

[7]Shenzhen Research Institute, City University of Hong Kong, Shenzhen 518057, China

***Corresponding authors.** E-mails:

kmyleung@cityu.edu.hk; zhiyzeng@cityu.edu.hk; qiujw@hkbu.edu.hk; b.h@cityu.edu.hk

†Equally contributed to this work.

Contents

1. Methods

2. Supplementary Figures 1-28

Figure S1. Schematic illustration of the electrochemical lithium-ion intercalation–assisted exfoliation method

Figure S2. SEM image of bulk MoS_2 powder

Figure S3. XRD spectra of bulk MoS_2 powder

Figure S4. Zetapotential of exfoliated MoS_2 NSs solution

Figure S5. SEM images of M1, M2, and M3 membrane surface

Figure S6. AFM images of M1, M2, and M3 membrane surface

Figure S7. Photos of the four aquarium tanks housing coral fish

Figure S8. Workflow for eDNA collection, processing, and analysis

Figure S9. Rarefaction curves for the samples from aquarium tests, mock community assays, and field tests

Figure S10. Adsorption curves fitted to the Thomas model of M0 and M1 membranes

Figure S11. Geographical location of the field sampling site

Figure S12. Flow rates of artificial seawater through M0, M1, M2, and M3 membranes during vacuum filtration

Figure S13. DNA yield through extracting the M0, M1, M2, and M3 membranes using two different methods

Figure S14. XPS spectra of M1 membrane after eDNA sampling

Figure S15. XPS spectra of M1 membrane before eDNA sampling

Figure S16. XPS spectra of M0 membrane after eDNA sampling

Figure S17. Assessing DNA quantity and quality extracted from M0 and M1 membranes

Figure S18. Cross-sectional SEM-EDX mapping of the M1 membrane after eDNA filtration

Figure S19. XPS depth profile of the M1 membrane after filtering the eDNA solution

Figure S20. XRD spectra of M1 membrane after eDNA sampling

Figure S21. Cross-sectional SEM image of M1 membrane after eDNA sampling

Figure S22. Raman spectra of M1 membrane after eDNA sampling

Figure S23. Mo release from M1 membrane during the seawater filtration

Figure S24. XPS spectra of M1 membrane during the seawater filtration

Figure S25. Scalable preparation of MoS_2 NSs

Figure S26. The most stable binding configurations of DNA bases on the 1T′-MoS_2

Figure S27. The adsorption configurations of DNA bases in parallel configurations after geometry optimizations with solvent environments

Figure S28. The adsorption energy comparisons of DNA bases for parallel configurations with/without solvent effect

3. Supplementary Tables 1-6

Table S1. Raw data statistics for eDNA assays

Table S2. Filtered community data for aquarium tests

Table S3. Detection probability of fish taxa in the mock community assays

Table S4. Filtered community data for the mock community test

Table S5. Filtered community data for the field test

Table S6. Quantitative cost analysis of the scalable preparation of MoS_2

Methods:

Preparation of MoS_2 nanosheets and corresponding nanolaminates. MoS_2 nanosheets were prepared using an electrochemical lithium-ion (Li^+) intercalation-assisted exfoliation method (see **Figure S1**).[1-3] The process began with Li^+ ions being intercalated into the interlayer spaces of bulk MoS_2 (4 mg) under galvanostatic discharge conditions in a coin cell. This step was performed with a current of 0.05 mA and a cutoff voltage of 0.9 V. Following the intercalation, the lithiated MoS_2 was exfoliated by sonicating the sample in 10 mL of deionized (DI) water. The intercalated Li^+ ions expanded the interlayer spacing, reducing the van der Waals forces that bonded the layers. Simultaneously, hydrogen gas generated during sonication further enhanced the exfoliation process, allowing it to complete within 5–10 minutes. The exfoliated nanosheets were then thoroughly purified through multiple rinses with DI water. Careful handling of any unreacted lithium foil is essential when disassembling the coin cell to ensure safety. The purified MoS_2 nanosheets were redispersed in DI water and vacuum-filtered onto a porous polymer substrate (MCE, 450 nm pore size, 50 mm diameter), forming an MoS_2 nanolaminates.[4] The thickness was precisely controlled by adjusting the mass of filtrated MoS_2 nanosheets.

Structural characterization of MoS_2 nanosheets and corresponding nanolaminates. The following characterization techniques were employed: SEM (Thermo Scientific Quattro S, operated at 20 kV), TEM (Tecnai G2 Spirit Twin, with an accelerating voltage of 120 kV), ADF-STEM (JEOL ARM200F spherical aberration-corrected transmission electron microscope, operated at 200 kV), XPS (Thermo Scientific K-Alpha Nexsa, Al Kα source, with binding energy calibrated using the C 1*s* peak at 284.8 eV), Raman (WITEC 300R Raman system, 532 nm), XRD (D2 PHASER XE-T, Cu Kα radiation source, 30 kV voltage and 10 mA current), AFM (Dimension 3100, Veeco, CA), Zeta potential (Malvern Zetasizer Nano series), and contact angle (OCA 15EC Contact Angle Tester).

Laboratory and field water sampling. Laboratory and field experiments were

conducted to evaluate the effect of MoS_2 membrane coatings on the efficiency of eDNA metabarcoding for detecting marine fish. In the aquarium trials, 1 L of artificial seawater was collected and combined from four separate tanks housing coral reef fish species, including Eyestripe Surgeonfish (*Acanthurus dussumieri*), Blue Devil (*Chrysiptera cyanea*), Threespot Dascyllus (*Dascyllus trimaculatus*), Stripey (*Microcanthus strigatus*), and Blue Tang (*Paracanthurus hepatus*) (**Figure S7**). The mixed water samples were filtered through MCE membranes coated with varying thicknesses of MoS_2 NSs (M1, M2, M3) as well as an uncoated membrane (M0). DNA was immediately extracted from the membranes following filtration.

For field tests, water samples were collected from the Hoi Ha Wan Marine Park, Hong Kong (GPS: 22.4675473, 114.3301480). Three replicates of 4 L seawater were obtained from a randomly selected site using a Niskin sampler and filtered *in situ* onto the MoS_2-coated or uncoated membranes (M0 and M1). The membranes were immediately placed in separate sterile Petri dishes, stored on ice, and subsequently transferred to a −80 °C freezer upon arrival at the laboratory. DNA extraction was performed the next day after sampling.

Mock community metabarcoding assay. A mock community assay was conducted to determine the detection limit of the M0 and M1 membranes on different amounts of fish DNA. We extracted tissue DNA from various fish specimens listed in **Table S3** using the CTAB-phenol-chloroform DNA extraction protocol. The fish DNA was then diluted to specific concentrations and mixed well in sterilised MilliQ water. The DNA mix was filtered using M0 and M1 membranes at a flow rate of 0.002 L min^{-1} using a syringe injector attached to a Teflon membrane holder. The membranes were then blown to dryness using the syringe to prevent liquid samples from remaining on the surface. The membranes were immediately subjected to further processing. We conducted ten biological replicates for each of the membranes, and the detection probability of the fish taxa was calculated by dividing the number of occurrences by the total sample size.

DNA adsorption assay. DNA adsorption was conducted to determine the DNA adsorption capacity of M0 and M1 membranes. We prepared a large amount of fish DNA from the muscle tissue of market-sold frozen mackerels using the CTAB-phenol-chloroform protocol. The concentration and quality of fish DNA were determined using a Nanodrop spectrophotometer, and then diluted with sterilised MilliQ water. The solution was filtered using M0 and M1 membranes, with a syringe injector attached to a Teflon membrane holder, at a flow rate of 0.002 L min^{-1} under a room temperature of 20 °C. The flow-through was collected using a sterile 15 mL tube, with samples taken every minute for the first 30 mL and then every 5 minutes until a total of 100 mL was collected. The DNA concentrations of the flow through were again determined to construct the sorption breakthrough curves of the membranes. We fitted the data using the Thomas model, assuming the membranes behaved like fixed-bed columns. The weight of the membranes was 0.049 g. The assay was conducted for three independent replicates.

DNA extraction, polymerase chain reaction (PCR), and high-throughput sequencing. Based on the manufacturer's guidelines, the eDNA was extracted from the membranes using the DNeasy PowerSoil Pro Kit (Qiagen, Hilden, Germany). The quantity and quality of the DNA samples were assessed using Qubit dsDNA-HS Fluorescence Assay Kit (Invitrogen, Carlsbad, CA, USA). The purity of the samples was determined by spectrophotometry, measuring the A260/280 absorbance ratio. Moreover, to determine the ssDNA/dsDNA ratio, we also measured the ssDNA concentrations using the Qubit ssDNA Fluorescence Assay Kit (Invitrogen, Carlsbad, CA, USA). The GC ratio per sequence of the samples was inferred from the sequencing results as visualized by the FastQC software package. For the fragment length distribution, we performed gel electrophoresis on the raw DNA samples and captured band intensities using a gel documentation system. The captured images were color-inverted and analyzed using ImageJ software. After quality inspections, we conducted PCR runs using 12S-V5 primer designated for vertebrate detection (F: AAGGCACTGGGATTAGATACCCC; R: AAGGCTAGAACAGGCTCCTCTAG)

(See **Figure S8** for details).[5] The PCR program comprised a 2-minute initial denaturation at 98 °C, followed by 35 cycles of denaturation at 98 °C for 10 seconds, annealing at 60 °C for 10 seconds, and extension at 68 °C for 5 seconds, culminating in a final extension at 68 °C for 2 minutes. The PCR products were checked for amplicon size and quantity before conducting paired-end 150 bp sequencing on a Novaseq 6000 platform, provided by a sequencing service company (Novogene Co., Ltd., Beijing, China).

Bioinformatics and ecological data analysis. The raw sequences were stripped of index and primer sequences using Cutadapt (version 4.8[6] and further processed within the Qiime 2 environment (version 2024.5).[7] We followed the same workflow as our previous study, including read merging, quality filtering, denoising, and taxonomic assignment of the amplicon sequence variants (ASVs).[8] To address potential contamination, we cleaned the data with laboratory controls. In addition, only reads from ray-finned fish (Class Actinopteri) were retained for subsequent analysis.

The ecological analysis was performed in R (ver. 4.4.1), mainly using the vegan package.[9,10] The raw community matrices were rarefied to the same sample size, and rarefaction curves were visualized to evaluate whether the sequencing depths were adequate. The rarefied community matrices were utilized to calculate fundamental biodiversity metrics, including ASVs richness, the Shannon H index, and evenness. In addition, nonmetric multidimensional scaling (NMDS) was performed using the Bray-Curtis distances of rarefied data, which were auto-transformed, limited to two dimensions, and 5000 maximum iterations. Pairwise comparisons were performed using Student's *t*-tests to assess potential statistically significant differences between biodiversity metrics.

Calculation Setup. In this work, the theoretical calculations are carried out through the density functional theory (DFT) based on the embedded CASTEP packages to explore the binding of DNA bases in 1T′-MoS_2.[11] In particular, we have utilized the generalized gradient approximation (GGA) and Perdew-Burke-Ernzerhof (PBE) functionals in this work to handle the exchange-correlation interactions.[12-14] The cutoff energy has been

set with ultrafine quality at 380 eV with the ultrasoft pseudopotentials. To achieve efficient energy minimizations, the k-point setting has been set to coarse quality and the Broyden-Fletcher-Goldfarb-Shannon (BFGS) algorithm is applied.[15] For the tests of solvent environment influences, we have simulated the seawater with a composition of 1 Na^+, 1 Cl^-, and 55 H_2O molecules, which has been introduced during the adsorption of DNA bases. For all the geometry optimizations, we have applied the following convergency criteria: 1) Hellmann-Feynman forces should not exceed 0.001 eV $Å^{-1}$, and 2) the total energy difference should not exceed 5×10^{-5} eV $atom^{-1}$.

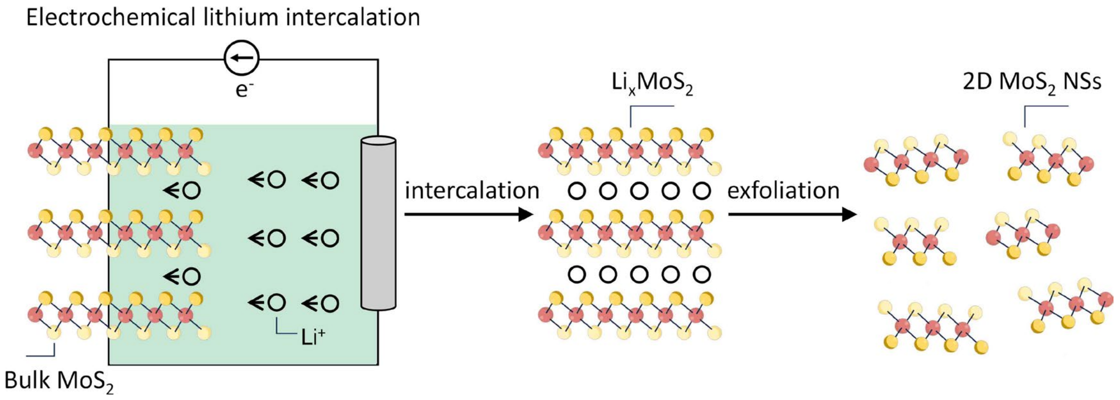

Figure S1. Schematic illustration of the electrochemical lithium-ion intercalation–assisted exfoliation method for the preparation of 2D MoS_2 NSs.

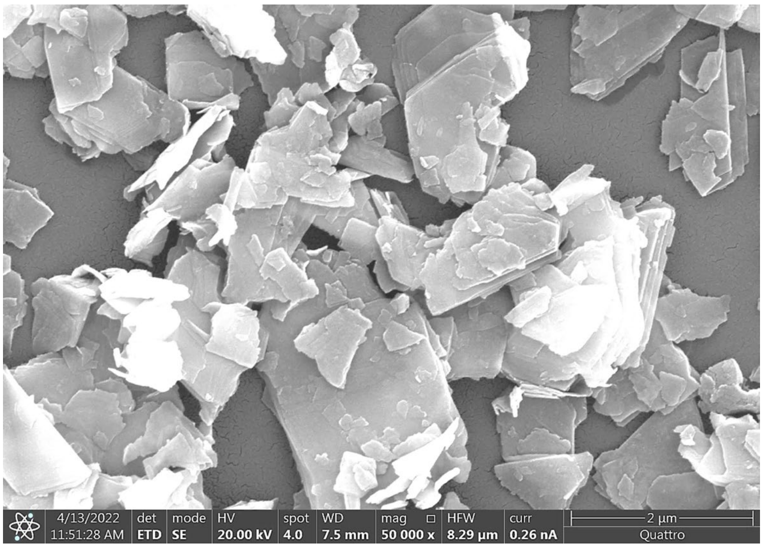

Figure S2. SEM image of bulk MoS_2.

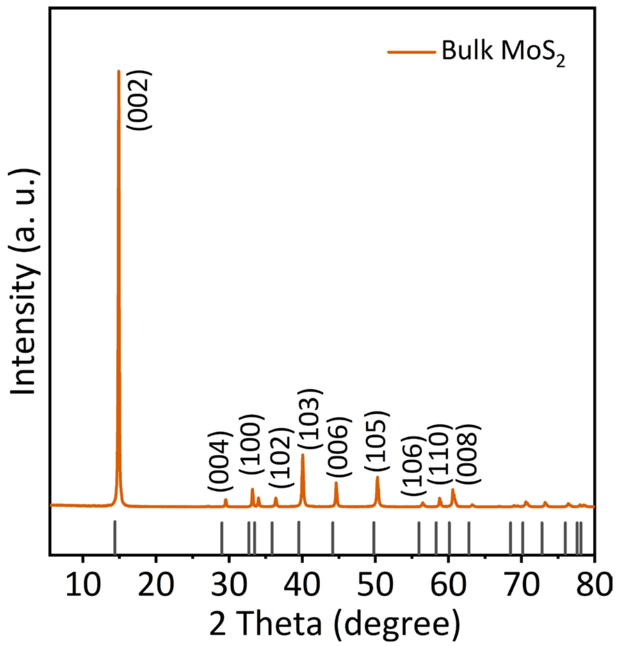

Figure S3. XRD spectra of bulk MoS_2.

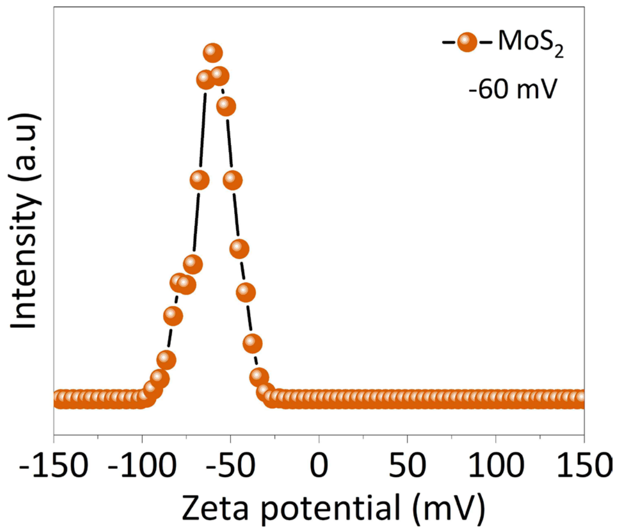

Figure S4. Zeta potential of exfoliated MoS_2 NSs.

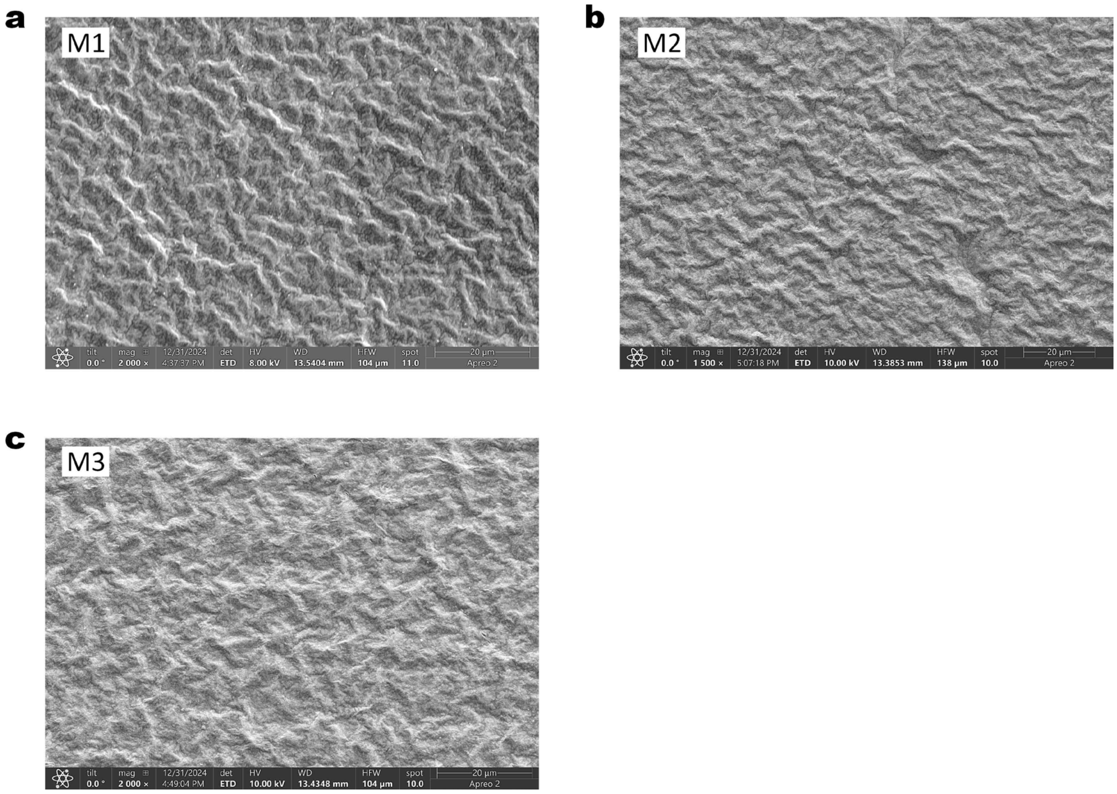

Figure S5. (**a**-**c**) SEM images (top view) of MoS_2 membranes surface (M1, M2, M3).

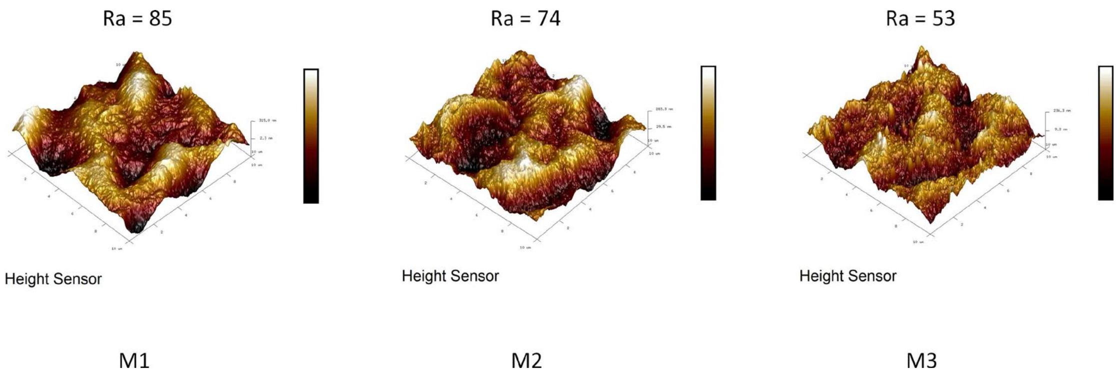

Figure S6. AFM images of MoS_2 membranes surface (M1, M2, M3).

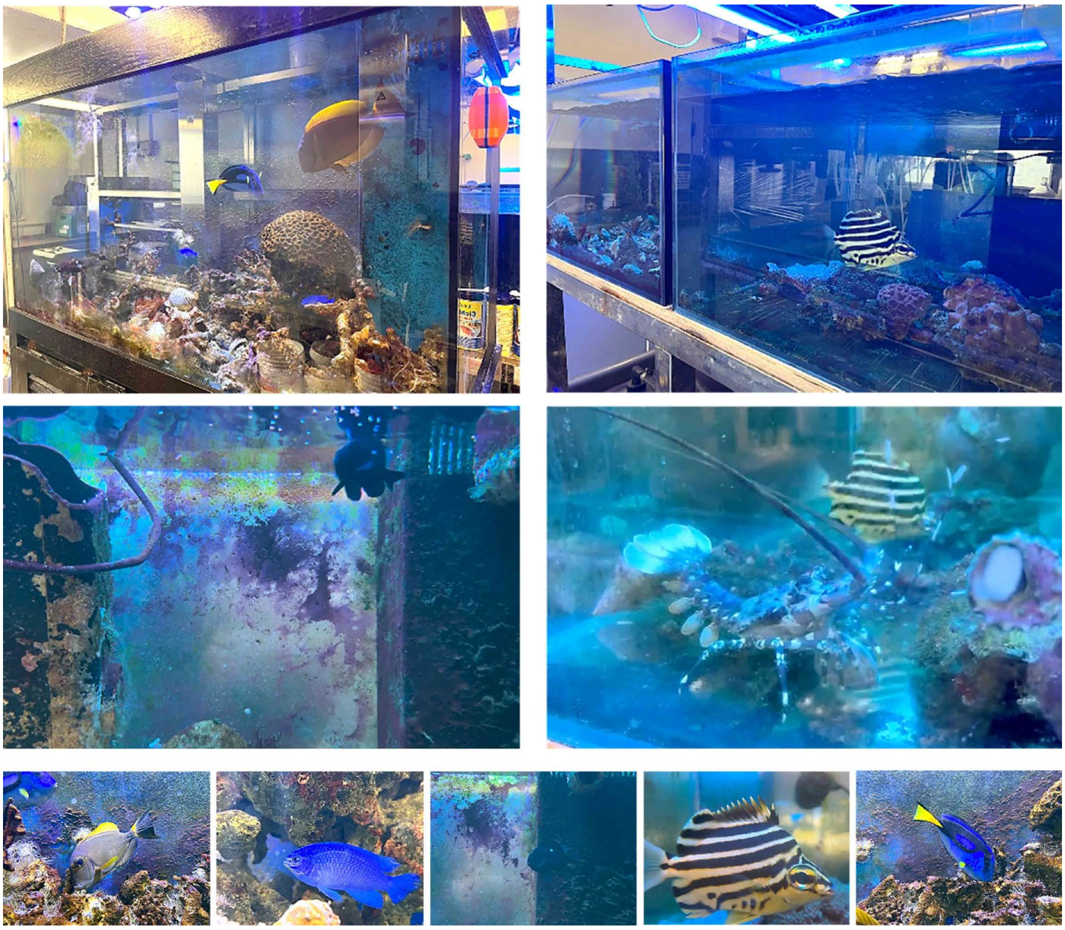

Figure S7. Photos of the four aquarium tanks housing coral fish and the original close-up photos.

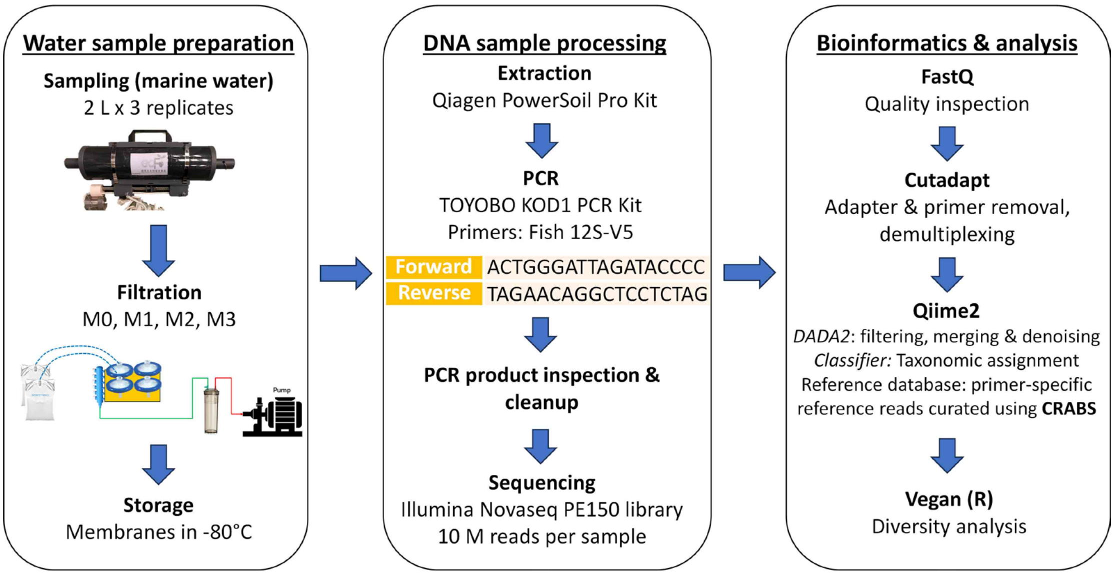

Figure S8. Workflow for eDNA collection, processing, and analysis.

Table S1. Raw data statistics for eDNA assays.

Data	ID	Input	Filtered	pass filter (%)	Denoised	Merged	merged (%)	Non-chimeric	non-chimeric (%)
aquarium	control	2930962	2894390	98.75	2893491	2854940	97.41	2842746	96.99
aquarium	M0	1049207	1036455	98.78	1036164	1026189	97.81	1015484	96.79
aquarium	M1	3126021	3089924	98.85	3089665	3059120	97.86	3036204	97.13
aquarium	M2	2254831	2226731	98.75	2226091	2205935	97.83	2180112	96.69
aquarium	M3	1181943	1167524	98.78	1167236	1156863	97.88	1136988	96.2
mock	M0_1	3923907	3852963	98.19	3852033	3698789	94.26	3601449	91.78
mock	M0_2	141552	138916	98.14	138593	135473	95.71	132781	93.8
mock	M0_3	2628622	2585862	98.37	2585271	2494468	94.9	2430890	92.48
mock	M0_4	1284846	1262975	98.3	1262424	1211735	94.31	1182158	92.01
mock	M0_5	1787641	1753412	98.09	1752925	1695145	94.83	1647527	92.16
mock	M0_6	2496570	2453349	98.27	2451941	2368710	94.88	2302209	92.21
mock	M0_7	1125845	1106154	98.25	1105720	1074185	95.41	1046312	92.94
mock	M0_8	125886	123841	98.38	123570	119666	95.06	117193	93.09
mock	M0_9	485649	477179	98.26	476920	460544	94.83	450476	92.76
mock	M0_10	177418	174597	98.41	174283	169710	95.66	166591	93.9
mock	M1_1	1910309	1874588	98.13	1873547	1789417	93.67	1736249	90.89
mock	M1_2	390356	383048	98.13	382671	368653	94.44	360035	92.23
mock	M1_3	1632346	1604836	98.31	1603907	1548431	94.86	1505756	92.24
mock	M1_4	1199965	1178801	98.24	1178048	1133303	94.44	1100486	91.71
mock	M1_5	1465251	1436576	98.04	1435467	1381295	94.27	1343560	91.69
mock	M1_6	1720562	1689555	98.2	1687166	1626166	94.51	1574011	91.48
mock	M1_7	1196009	1174414	98.19	1173633	1134438	94.85	1104958	92.39
mock	M1_8	286754	281951	98.33	281469	266395	92.9	260177	90.73
mock	M1_9	754239	741041	98.25	740310	703655	93.29	687914	91.21
mock	M1_10	468010	460752	98.45	460395	444428	94.96	434117	92.76
field	fcon	1902	1843	96.9	1834	1834	96.42	1834	96.42

field	lcon	4018	3932	97.86	3911	3896	96.96	3896	96.96
field	M0-1	1530665	1509986	98.65	1509567	1487410	97.17	1477433	96.52
field	M0-2	1928715	1904741	98.76	1904531	1878634	97.4	1871438	97.03
field	M0-3	1404717	1386646	98.71	1386415	1366767	97.3	1359804	96.8
field	M2-1	1811015	1785360	98.58	1784784	1760641	97.22	1749227	96.59
field	M2-2	1498738	1476241	98.5	1475766	1457340	97.24	1444597	96.39
field	M2-3	1997170	1968307	98.55	1968032	1941646	97.22	1929530	96.61

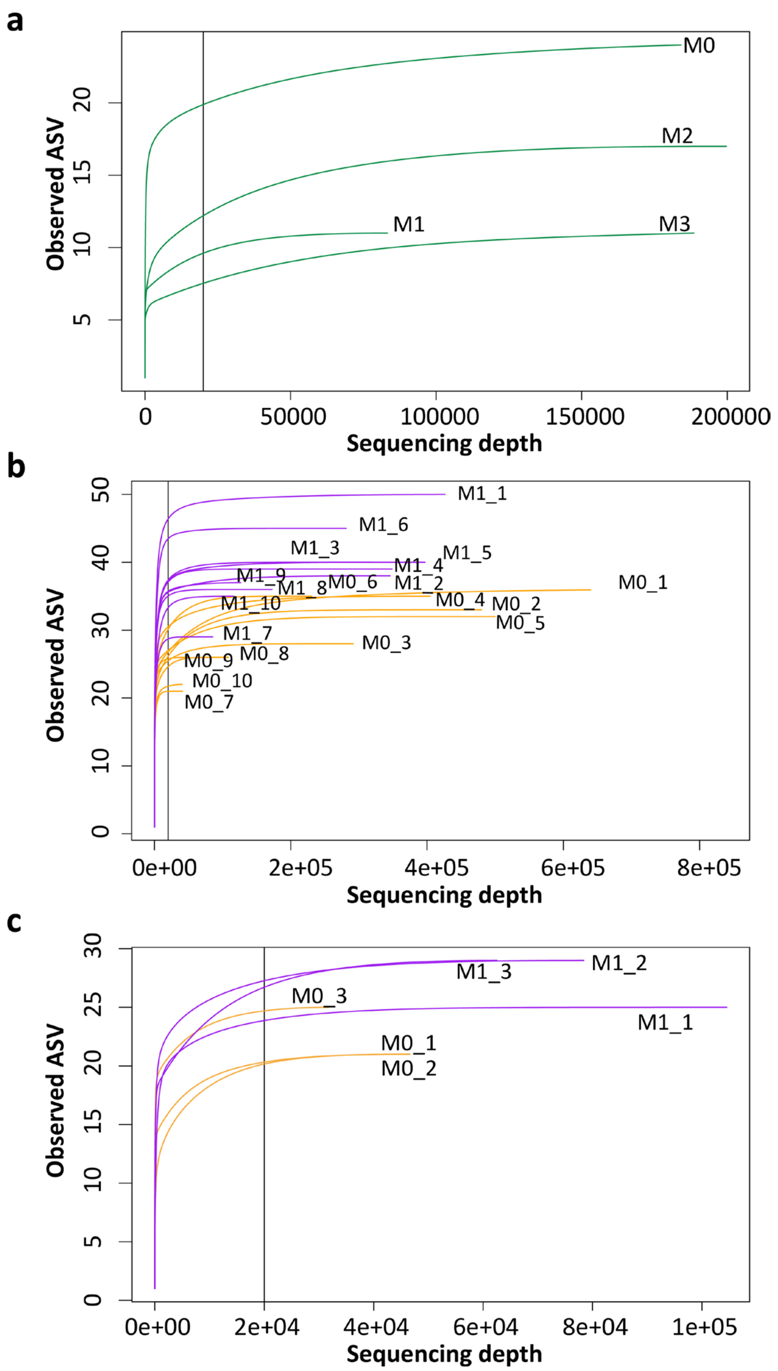

Figure S9. Rarefaction curves for the samples from (**a**) aquarium tests, (**b**) mock community assays, and (**c**) field tests.

Table S2. Filtered community data for aquarium tests.

Order	Family	Genus	Taxa	M0	M1	M2	M3
Acanthuriformes	Acanthuridae	*Acanthurus*	*Acanthurus dussumieri*	18992	11473	15159	10229
Carangiformes	Carangidae	*Alepes*	*Alepes* sp.	0	7	2	0
nan	Ambassidae	*Ambassis*	*Ambassis gymnocephalus*	300	0	0	0
nan	Pomacentridae	*Chrysiptera*	*Chrysiptera cyanea*	6740	3547	10087	6507
Pleuronectiformes	Cynoglossidae	*Cynoglossus*	*Cynoglossus melampetalus*	21	0	0	0
nan	Pomacentridae	*Dascyllus*	*Dascyllus* sp.	3530	4384	6929	1950
Gobiiformes	Gobiidae	*Favonigobius*	*Favonigobius* sp. CBM:ZF:19041	0	0	0	0
Clupeiformes	Pristigasteridae	*Ilisha*	*Ilisha elongata*	151	0	0	0
nan	Sciaenidae	*Johnius*	*Johnius carouna*	658	0	0	0
nan	Sciaenidae	*Johnius*	*Johnius belangerii*	0	4	5	2
Clupeiformes	Clupeidae	*Konosirus*	*Konosirus punctatus*	0	0	30	0
nan	Sciaenidae	*Larimichthys*	*Larimichthys crocea*	836	0	0	2
Lutjaniformes	Lutjanidae	*Lutjanus*	*Lutjanus argentimaculatus*	0	0	0	0
Centrarchiformes	Kyphosidae	*Microcanthus*	*Microcanthus strigatus*	23405	46557	31753	48175
Tetraodontiformes	Monacanthidae	*Monacanthus*	*Monacanthus chinensis*	442	4	3	0
Mugiliformes	Mugilidae	*Mugil*	*Mugil cephalus*	1	0	1	0
Mugiliformes	Mugilidae	*Mugil*	*Mugil curema*	110	0	0	0
Clupeiformes	Clupeidae	*Nematalosa*	*Nematalosa japonica*	954	0	1	0
Lutjaniformes	Haemulidae	*Orthopristis*	*Orthopristis chrysoptera*	20	559	114	106
Acanthuriformes	Acanthuridae	*Paracanthurus*	*Paracanthurus hepatus*	25084	16036	18609	16248
Chaetodontiformes	Leiognathidae	*Photopectoralis*	*Photopectoralis bindus*	2	0	3	0
Perciformes	Platycephalidae	*Platycephalus*	*Platycephalus* sp.	0	0	45	0
Siluriformes	Plotosidae	*Plotosus*	*Plotosus* sp.	1	2	0	0
Salmoniformes	Salmonidae	*Salmo*	*Salmo salar*	454	0	474	0
Clupeiformes	Clupeidae	*Sardinella*	*Sardinella* sp.	711	649	0	0
nan	Siganidae	*Siganus*	*Siganus* sp.	3	0	5	2
Pleuronectiformes	Soleidae	*Solea*	*Solea ovata*	0	0	0	0
Clupeiformes	Engraulidae	*Stolephorus*	*Stolephorus insularis*	684	0	0	0
Mugiliformes	Mugilidae	*Valamugil*	*Valamugil speigleri*	123	0	2	1

Table S3. Detection probability of fish taxa in the mock community assays.

Taxa	Input DNA (ng)	Detection probability		*t*-test
		M0	M1	
Pagrus major	0.1	0.6	1.0	0.037
*Echeneis naucrates**	0.1	0.1	0.9	0.003
Sparus aurata	0.1	0.6	1.0	0.037
Leiognathus equula	0.1	0.7	1.0	0.081
Scyris indica	1	0.9	1.0	0.343
Neopomacentrus bankieri	1	0.9	1.0	0.343
Arothron hispidus	1	1.0	1.0	
*Apogonichthyoides taeniatus**	10	1.0	1.0	
Monacanthus chinensis	10	1.0	1.0	
Sillago Aeolus	10	1.0	1.0	
*Amblyeleotris gymnocephala**	50	1.0	1.0	
Cephalopholis boenak	50	1.0	1.0	
Platycephalus sp. 1 LL-2021	50	1.0	1.0	
*Acanthopagrus schlegelii**	100	1.0	1.0	
Terapon jarbua	100	1.0	1.0	
*Rhabdosargus sarba**	100	1.0	1.0	

*Species name inferred from taxa annotated to only genus/family levels.

Table S4. Filtered community data for the mock community test.

Taxa	M0									
	1	2	3	4	5	6	7	8	9	10
Pagrus major	18	97	1049	0	448	464	6	0	0	0
*Echeneis naucrates**	0	0	0	0	1001	0	0	0	0	0
Sparus aurata	13	0	730	160	380	743	1364	0	0	0
Leiognathus equula	18	0	263	640	239	1219	0	0	6	9
Scyris indica	1197	20	12	6	818	750	10	116	325	0
Neopomacentrus bankieri	682	239	1194	825	21	657	879	138	352	0
Arothron hispidus	2883	204	2142	778	2534	1499	985	140	544	132
*Apogonichthyoides taeniatus**	9007	188	3709	1393	2645	1641	1379	534	588	531
Monacanthus chinensis	97049	3685	34617	26407	30858	32999	23604	4920	7490	4519
Sillago aeolus	23618	1197	14049	8699	8206	8306	4815	1003	4065	684
*Amblyeleotris gymnocephala**	51814	3072	35259	25131	34503	50817	21105	2825	6161	3443
Cephalopholis boenak	58156	2078	24804	16944	21883	34303	18320	2371	5142	2242
Platycephalus sp. 1 LL-2021	64505	5613	37781	26693	29021	40466	26407	4898	7667	5671
*Acanthopagrus schlegelii**	49731	2204	23026	17931	20347	26134	10954	1837	6359	2795
Terapon jarbua	211160	7845	121399	85831	108444	114822	49468	11349	35833	12107
*Rhabdosargus sarba**	212805	12860	145759	78515	133007	162070	63803	11324	31066	16187
% target fish	86.7	90.0	85.9	90.7	89.2	86.0	88.1	92.8	91.9	90.0

Taxa	M1									
	1	2	3	4	5	6	7	8	9	10
Pagrus major	363	23	777	363	277	398	87	140	131	150
*Echeneis naucrates**	95	24	67	176	0	56	135	19	62	49
Sparus aurata	482	122	301	270	208	327	194	79	173	44
Leiognathus equula	65	68	134	352	317	86	50	17	167	65
Scyris indica	449	140	253	240	201	174	307	27	166	155
Neopomacentrus bankieri	609	257	869	857	563	999	790	300	598	411
Arothron hispidus	3577	664	2581	2287	2596	2562	2059	629	1133	1122
*Apogonichthyoides taeniatus**	4803	853	3751	2198	3775	5534	3203	841	1867	1265
Monacanthus chinensis	54326	11215	34035	33062	39840	43118	27205	9132	19174	13185
Sillago aeolus	8165	3233	5020	6533	6761	8236	5274	2165	3667	3087
*Amblyeleotris gymnocephala**	44899	10544	36600	29881	37934	44898	27317	7878	18877	12616
Cephalopholis boenak	38684	7833	27518	22097	21176	29946	22712	6481	9723	9258
Platycephalus sp. 1 LL-2021	40384	15381	40054	38287	44499	48223	36028	7651	18270	16639
*Acanthopagrus schlegelii**	25635	6305	21192	19633	20442	23051	16046	3782	10492	6373
Terapon jarbua	84314	22687	67237	60169	68968	80297	52083	18383	35285	23116
*Rhabdosargus sarba**	107060	32945	92506	86766	94049	99459	70863	26020	48357	34115
% target fish	86.7	88.1	86.1	86.0	87.4	86.4	84.6	88.7	86.7	86.5

Table S5. Filtered community data for the field test.

Order	Family	Genus	Taxa	fcon	lcon	M0-1	M0-2	M0-3	M2-1	M2-2	M2-3
Spariformes	Sparidae	*Acanthopagrus*	*Acanthopagrus* sp.	5	0	5	2	1203	3	317	901
Carangiformes	Carangidae	*Alepes*	*Alepes* sp.	0	0	0	0	7	0	91	3
nan	Ambassidae	*Ambassis*	*Ambassis buruensis*	0	0	0	13	1673	217	0	1028
nan	Ambassidae	*Ambassis*	*Ambassis gymnocephalus*	0	0	5	0	0	82	276	0
Atheriniformes	Atherinidae	*Atherinomorus*	*Atherinomorus lacunosus*	0	0	0	0	0	0	98	1
Syngnathiformes	Callionymidae	*Callionymus*	*Callionymus bairdi*	0	0	0	2	545	0	3	0
Cypriniformes	Cyprinidae	*Carassius*	*Carassius* sp.	0	0	0	0	0	0	0	0
Perciformes	Serranidae	*Cephalopholis*	*Cephalopholis boenak*	0	0	0	3	2	0	1	329
Carangiformes	Carangidae	*Decapterus*	*Decapterus maruadsi*	0	0	6	5	4	0	12	0
Gobiiformes	Gobiidae	*Drombus*	*Drombus* sp. KUROKO-HAZE	0	0	0	0	269	0	0	1
Elopiformes	Elopidae	*Elops*	*Elops* sp.	0	0	2	395	1762	66	5	915
Clupeiformes	Engraulidae	*Encrasicholina*	*Encrasicholina heteroloba*	0	0	0	0	0	14	0	0
Clupeiformes	Engraulidae	*Encrasicholina*	*Encrasicholina* sp.	36	3	1183	7668	5837	10174	4429	7682
Perciformes	Serranidae	*Epinephelus*	*Epinephelus* sp.	2	0	4	0	0	3	417	4
Chaetodontiformes	Leiognathidae	*Equulites*	*Equulites rivulatus*	0	0	0	0	0	0	188	2
Chaetodontiformes	Leiognathidae	*Gazza*	*Gazza minuta*	0	0	0	0	802	0	4	0
Gerreiformes	Gerreidae	*Gerres*	*Gerres* sp.	72	3	15679	117	2688	1253	13508	1057
Beloniformes	Hemiramphidae	*Hemiramphus*	*Hemiramphus depauperatus*	0	0	0	0	0	0	0	300
Clupeiformes	Pristigasteridae	*Ilisha*	*Ilisha elongata*	0	0	326	0	0	0	0	2
nan	Sciaenidae	*Johnius*	*Johnius belangerii*	0	0	0	0	0	0	0	0
nan	Sciaenidae	*Johnius*	*Johnius carouna*	0	0	0	0	0	9	0	0
Clupeiformes	Clupeidae	*Konosirus*	*Konosirus punctatus*	0	0	334	0	5	0	377	0
Tetraodontiformes	Tetraodontidae	*Lagocephalus*	*Lagocephalus* sp.	0	0	0	0	6	0	3	4
Tetraodontiformes	Tetraodontidae	*Lagocephalus*	*Lagocephalus spadiceus*	0	0	0	0	0	0	0	0
Lutjaniformes	Lutjanidae	*Lutjanus*	*Lutjanus argentimaculatus*	0	0	2	2	0	88	1	0
Tetraodontiformes	Monacanthidae	*Monacanthus*	*Monacanthus chinensis*	0	0	0	0	0	95	0	0
Mugiliformes	Mugilidae	*Moolgarda*	*Moolgarda perusii*	0	0	0	6	572	4	562	5
Mugiliformes	Mugilidae	*Mugil*	*Mugil cephalus*	0	0	2564	10651	4725	29	1242	5087
Mugiliformes	Mugilidae	*Mugil*	*Mugil curema*	0	0	319	0	0	1	0	0
Mugiliformes	Mugilidae	*Mugil*	*Mugil liza*	0	0	0	0	0	0	0	550
Clupeiformes	Clupeidae	*Nematalosa*	*Nematalosa japonica*	0	0	445	0	486	35	0	0
Clupeiformes	Clupeidae	*Nematalosa*	*Nematalosa nasus*	37	10	3188	3388	4072	11372	3830	3778
Clupeiformes	Clupeidae	*Nematalosa*	*Nematalosa* sp.	0	0	0	0	0	0	160	0
nan	Pomacentridae	*Neopomacentrus*	*Neopomacentrus bankieri*	0	0	0	0	0	0	138	0
Gobiiformes	Gobiidae	*Oxyurichthys*	*Oxyurichthys* sp.	7	11	2149	4795	2627	5357	693	4753
nan	Sciaenidae	*Pennahia*	*Pennahia aneus*	0	0	0	0	0	0	0	202
Chaetodontiformes	Leiognathidae	*Photopectoralis*	*Photopectoralis bindus*	3	0	306	0	0	88	316	7

Mugiliformes	Mugilidae	*Planiliza*	*Planiliza macrolepis*	0	0	0	119	416	0	0	3
Perciformes	Platycephalidae	*Platycephalus*	*Platycephalus indicus*	0	0	0	3	0	87	0	0
Siluriformes	Plotosidae	*Plotosus*	*Plotosus lineatus*	0	0	0	118	0	49	0	0
Siluriformes	Siluridae	*Pterocryptis*	*Pterocryptis cochinchinensis*	0	0	0	0	89	0	0	0
Clupeiformes	Clupeidae	*Sardinella*	*Sardinella* sp.	0	0	786	2316	863	488	1815	1089
Cypriniformes	Nemacheilidae	*Schistura*	*Schistura fasciolata*	0	0	0	0	0	0	0	263
Carangiformes	Carangidae	*Seriola*	*Seriola* sp.	0	0	0	0	0	0	0	164
nan	Siganidae	*Siganus*	*Siganus* sp.	2	0	274	1168	2	105	333	162
nan	Sillaginidae	*Sillago*	*Sillago aeolus*	0	0	279	33	2047	413	8	1636
Pleuronectiformes	Soleidae	*Solea*	*Solea ovata*	0	0	0	0	0	116	0	2
nan	Sphyraenidae	*Sphyraena*	*Sphyraena qenie*	0	0	0	0	0	0	56	0
Clupeiformes	Clupeidae	*Spratelloides*	*Spratelloides gracilis*	10	0	3904	955	718	1613	2853	1831
Clupeiformes	Engraulidae	*Stolephorus*	*Stolephorus indicus*	0	0	1	2	341	0	0	0
Clupeiformes	Engraulidae	*Thryssa*	*Thryssa* sp.	0	0	0	0	0	0	25	0

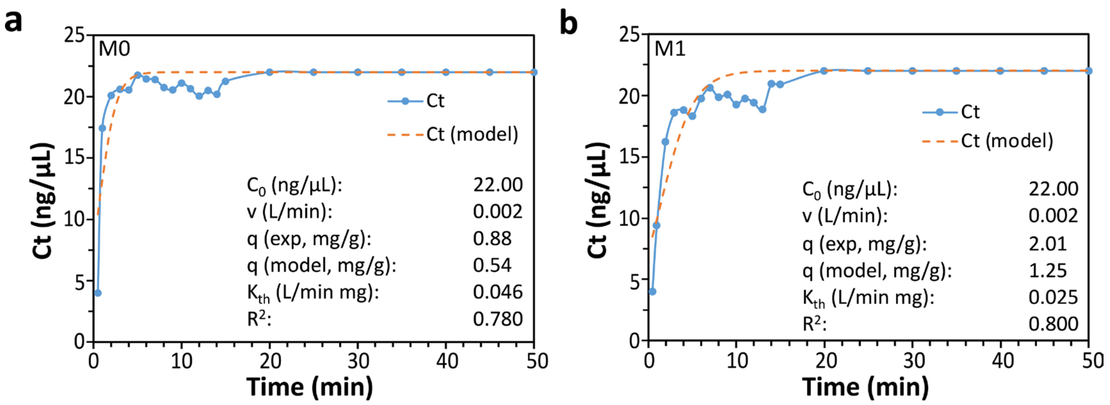

Figure S10. Adsorption curves fitted to the Thomas model of M0 and M1 membranes.

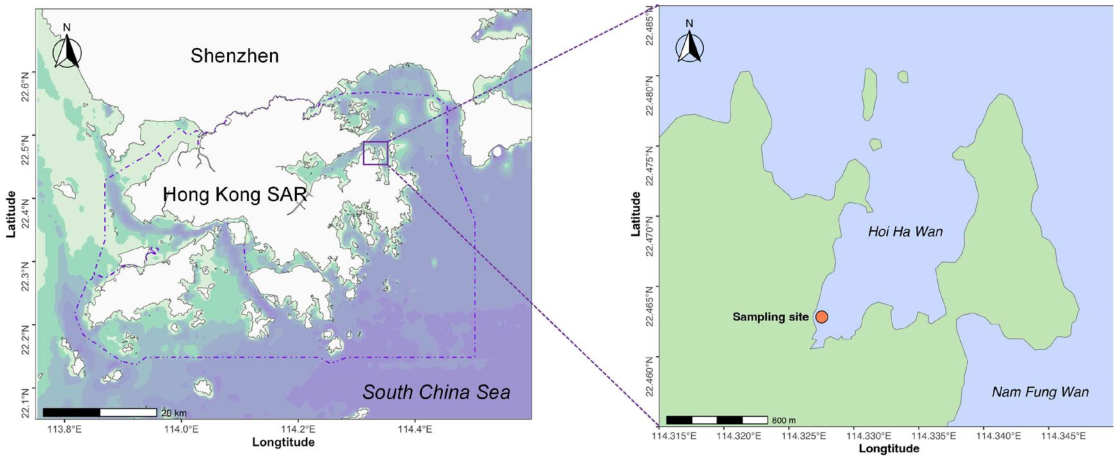

Figure S11. Geographical location of the field sampling site.

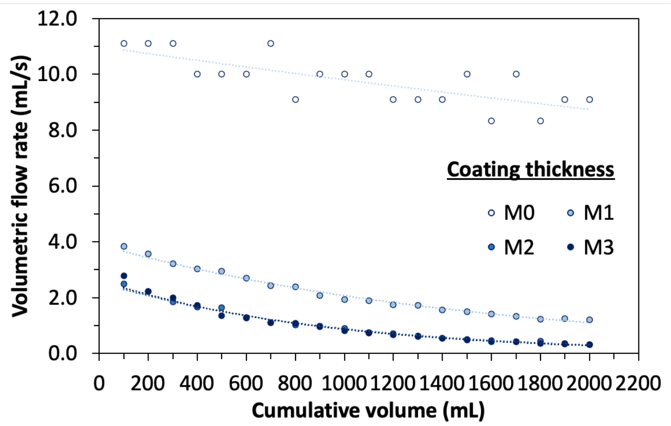

Figure S12. Flow rates of artificial seawater through MCE membranes coated with varying thicknesses of MoS_2 NSs (M0, M1, M2, M3) during vacuum filtration.

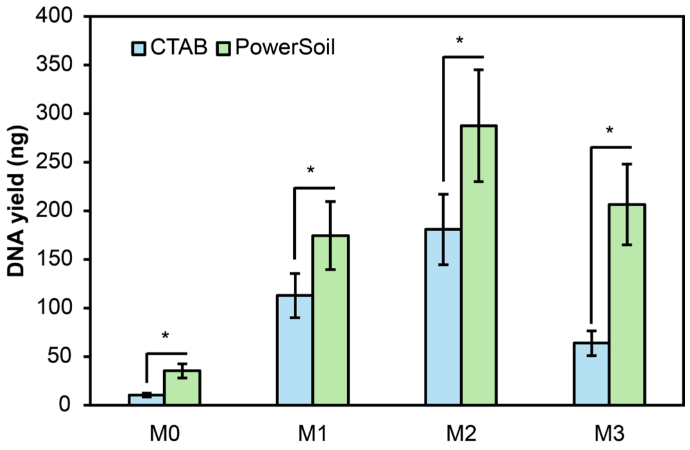

Figure S13. DNA yield from extracting M0, M1, M2, and M3 using two methods (CTAB-phenol-chloroform and PowerSoil kit).

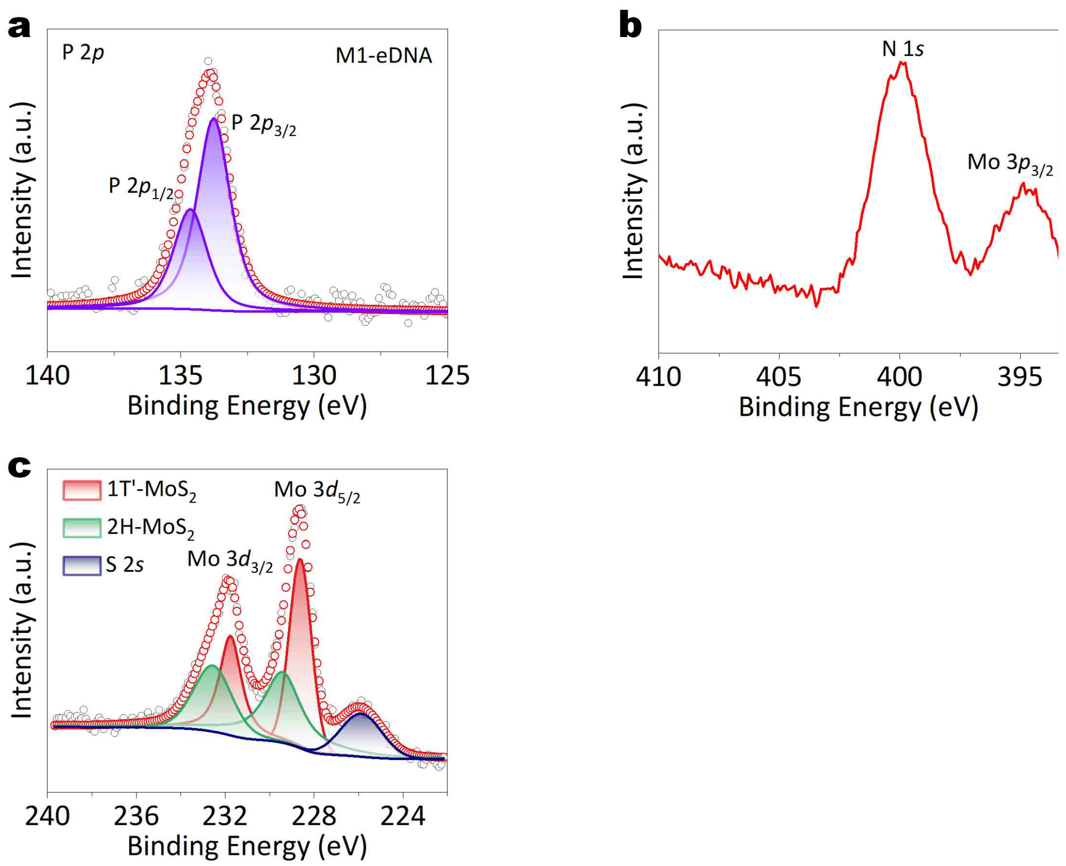

Figure S14. XPS spectra of M1 membrane after eDNA sampling: (**a**) P 2*p*; (**b**) N 1*s*; (**c**) Mo 3*d*.

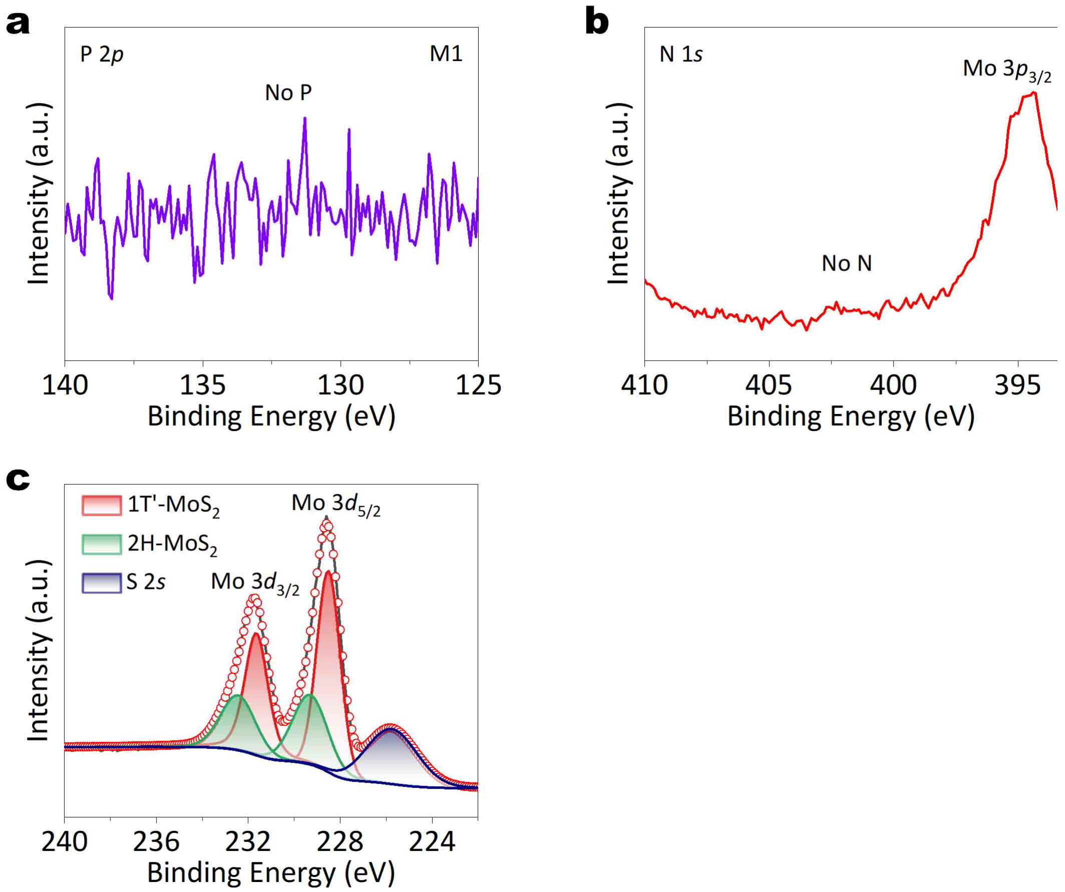

Figure S15. XPS spectra of M1 membrane before eDNA sampling: (**a**) P 2*p*; (**b**) N 1*s*; (**c**) Mo 3*d*.

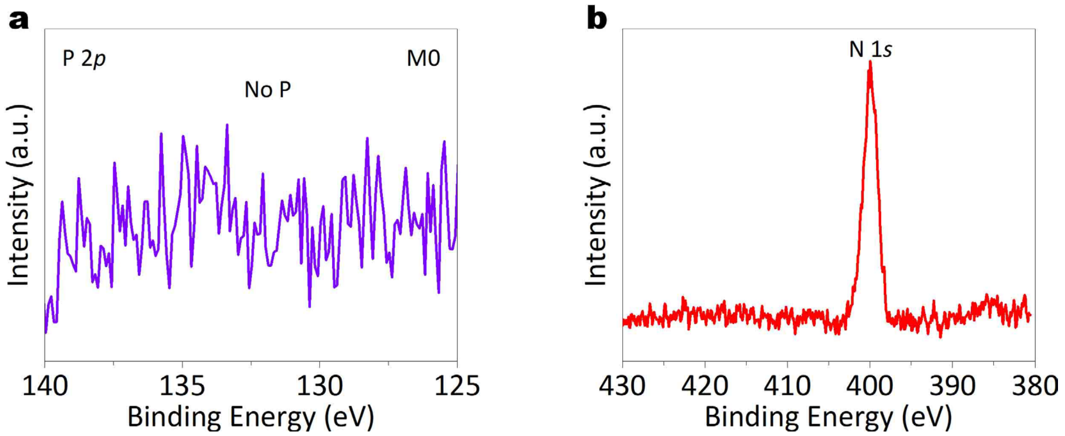

Figure S16. XPS spectra of M0 membrane after eDNA sampling: (**a**) P 2*p*; (**b**) N 1*s*.

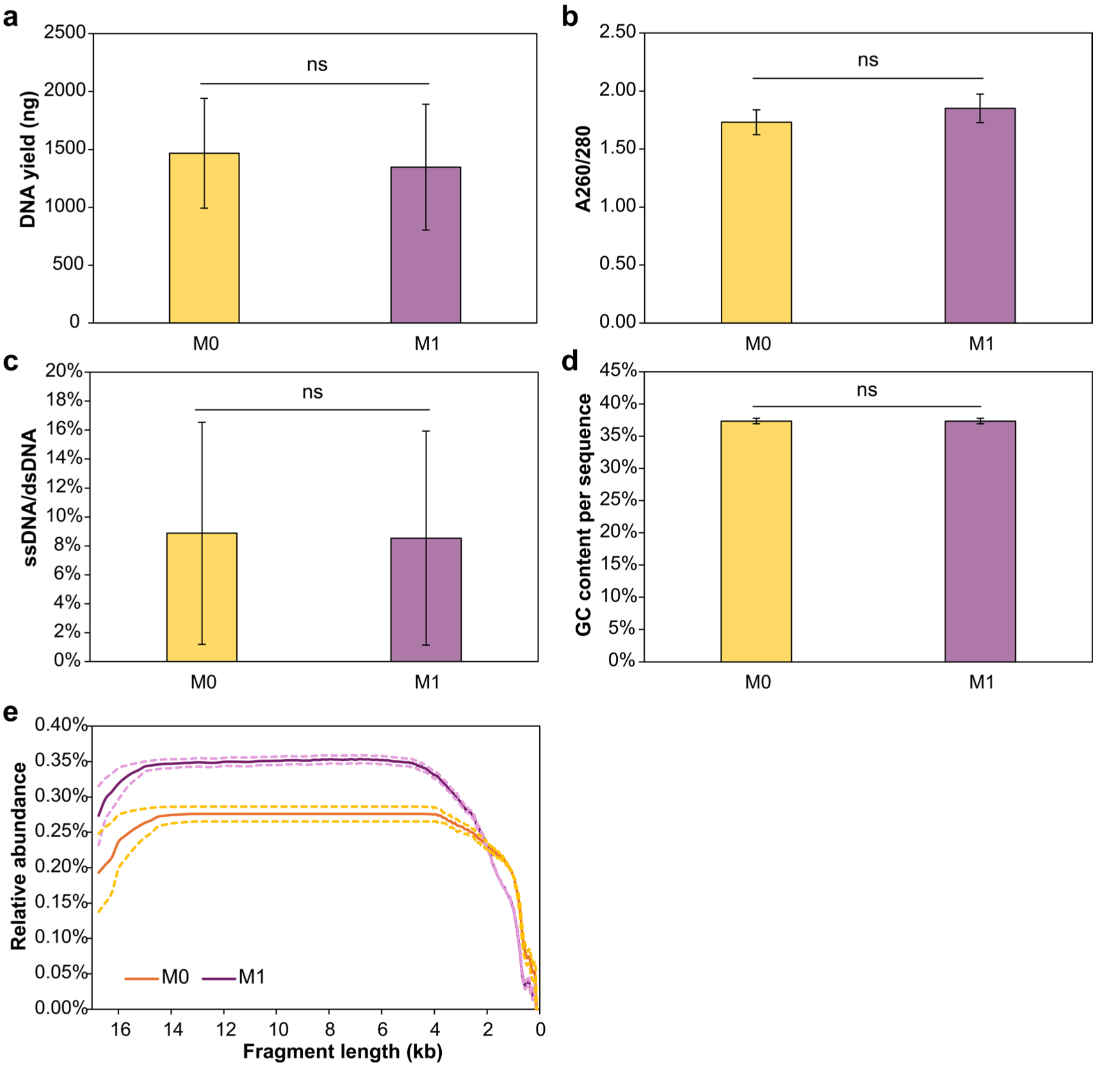

Figure S17. Assessing DNA quantity and quality extracted from M0 and M1 membranes.

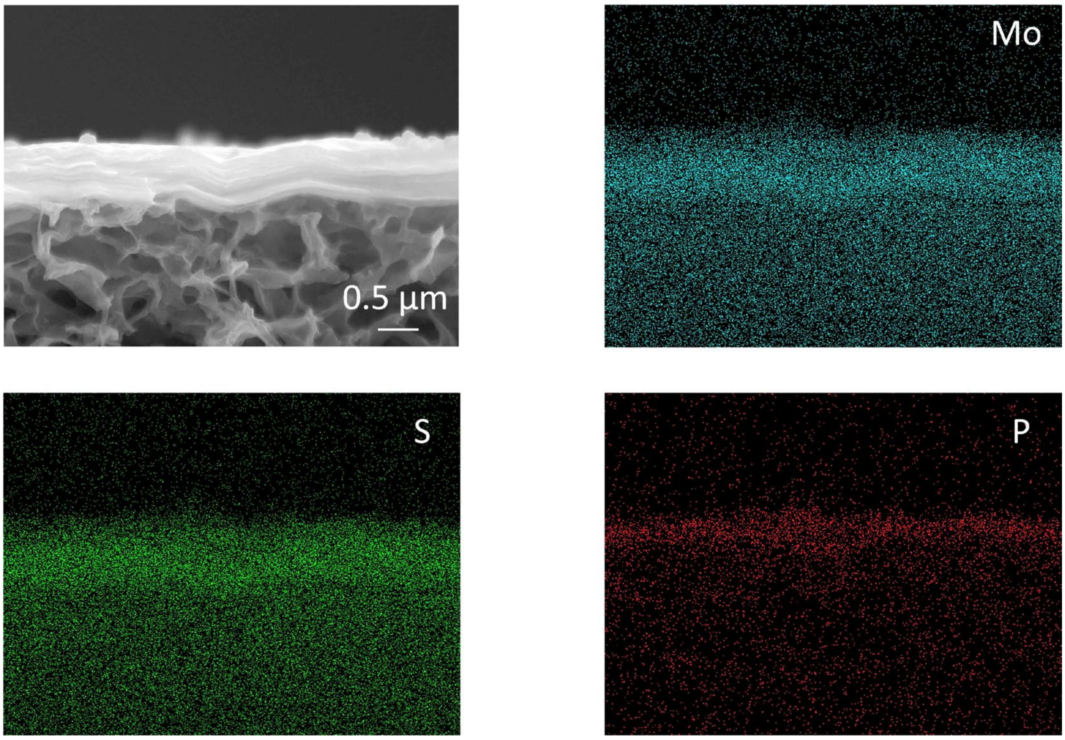

Figure S18. Cross-sectional SEM image of the M1 membrane after eDNA filtration, with corresponding EDX maps of the Mo, S, and P elements.

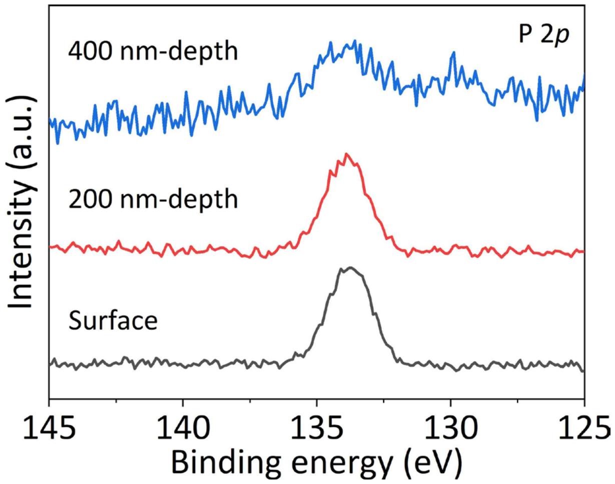

Figure S19. XPS depth profile of the M1 membrane after filtering the eDNA solution.

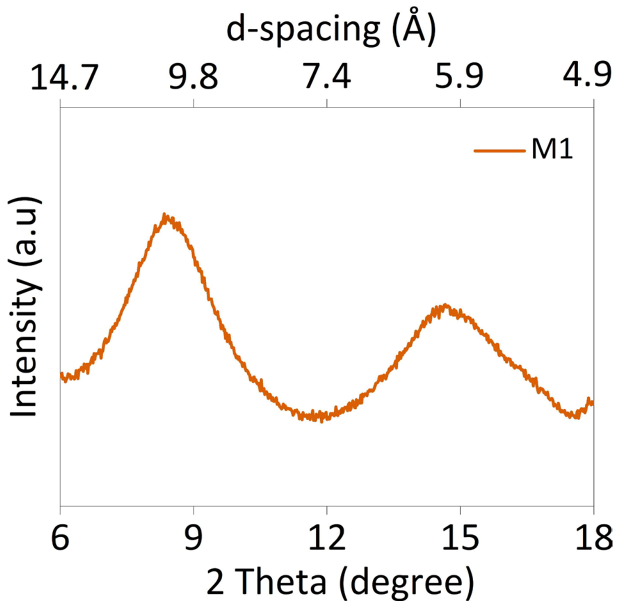

Figure S20. XRD spectra of M1 membrane after eDNA sampling.

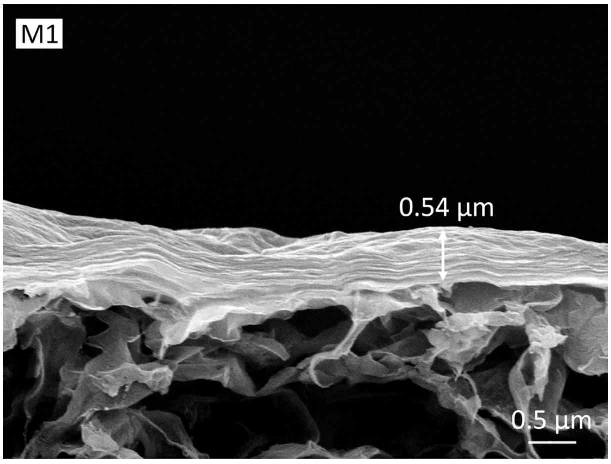

Figure S21. Cross-sectional SEM image of M1 membrane after eDNA sampling.

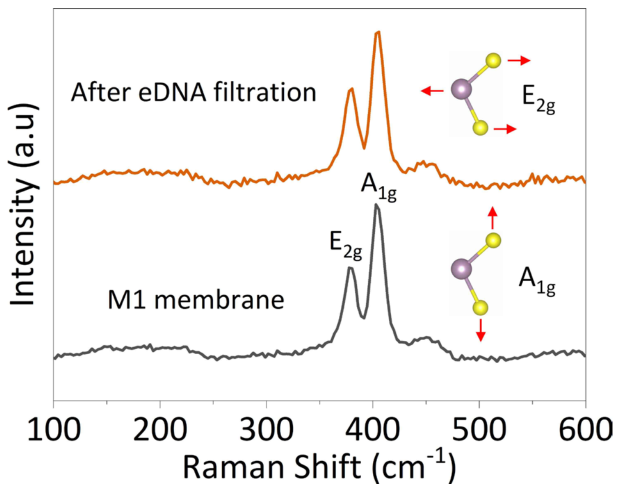

Figure S22. Raman spectra of M1 membrane after eDNA sampling.

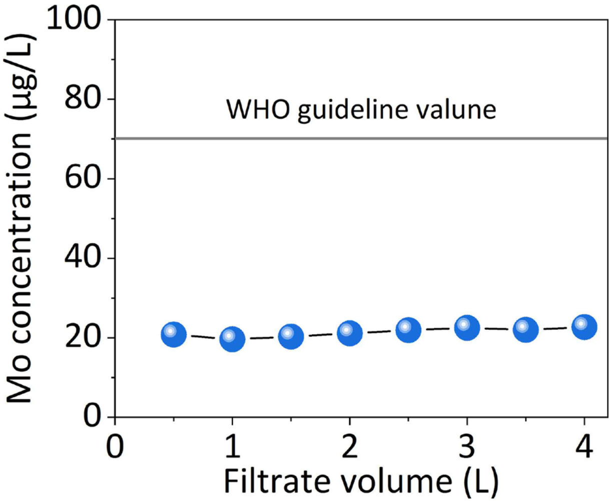

Figure S23. The concentration of released Mo from M1 membrane during the 4L seawater filtration. The WHO guideline value for Mo is 70 μg/L.

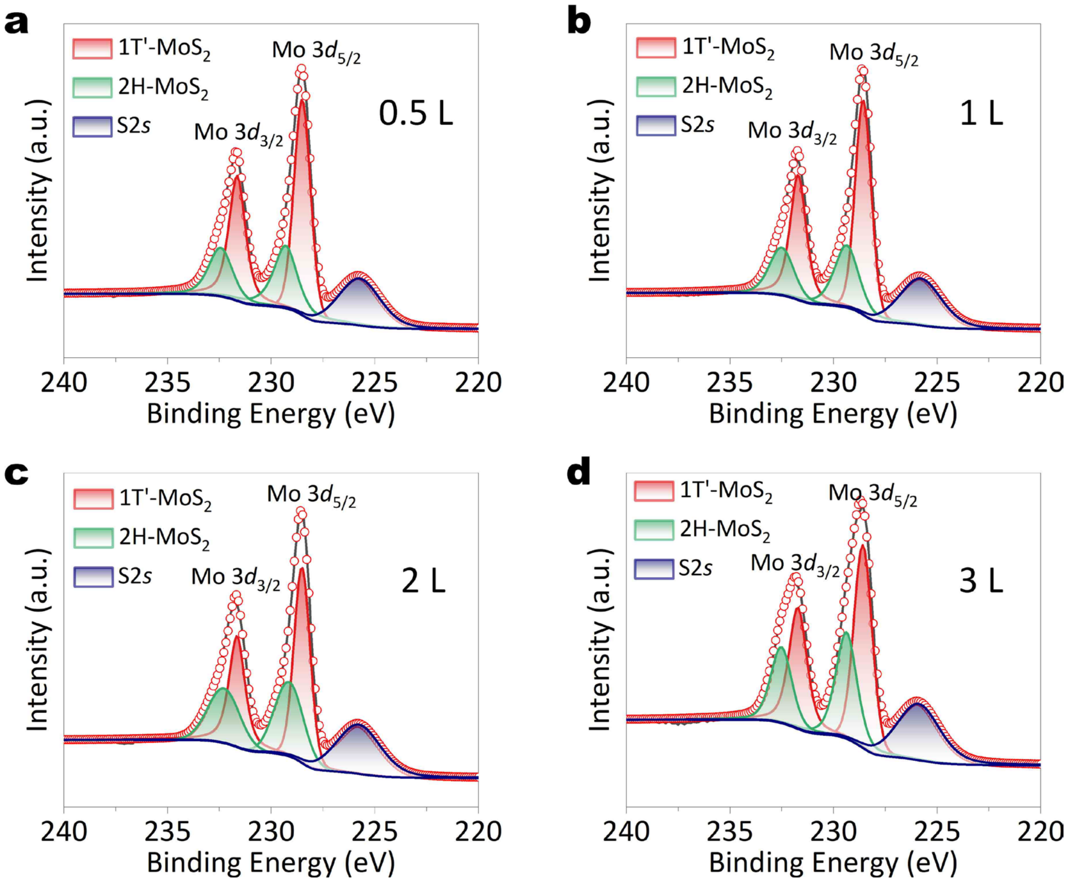

Figure S24. XPS Mo 3*d* spectra of M1 membrane after filtration of seawater volumes of 0.5 L, 1 L, 2 L, and 3 L.

Table S6. Quantitative cost analysis of the scalable preparation of MoS_2.

Required consumables and processes	Mass or other	Price
Pouch battery	1	28$ per
$LiFePO_4$ (Anode)	0.02 kg	14$ kg^{-1}
MoS_2 (Cathode)	0.01 kg	138$ kg^{-1}
Cu (current collector)	0.001 kg	10.5$ kg^{-1}
Al (current collector)	0.001 kg	2.7$ kg^{-1}
$LiPF_6$ in DC/EMC (Electrolyte)	0.001 kg	468$ kg^{-1}
Celgard 2300 (Separator)	0.045 m^2	159.4$ m^{-2}
Water	3 kg	0.8$ t^{-1}
Charging energy	0.016 kwh	0.05$ kwh^{-1}
Ultrasonic energy	0.05 kwh	0.05$ kwh^{-1}

The total cost was calculated based on 10g of bulk MoS_2 as the raw material, as shown below:

$$1\times28 + 0.02\times14 + 0.01\times138 + 0.001\times10.5 + 0.001\times2.7 + 0.001\times468 + 0.045\times159.4 + 3\times0.8\times10^{-3} + 0.016 \times 0.05 + 0.05 \times 0.05 = 37.32\$$$

Based on our experience, 10 g of bulk MoS_2 can yield 1 g of MoS_2 nanosheets, which is sufficient to fabricate approximately 500 membranes (diameter = 45 mm, thickness $\approx$ 500 nm), the estimated cost per MoS_2 membrane is about 0.075$.

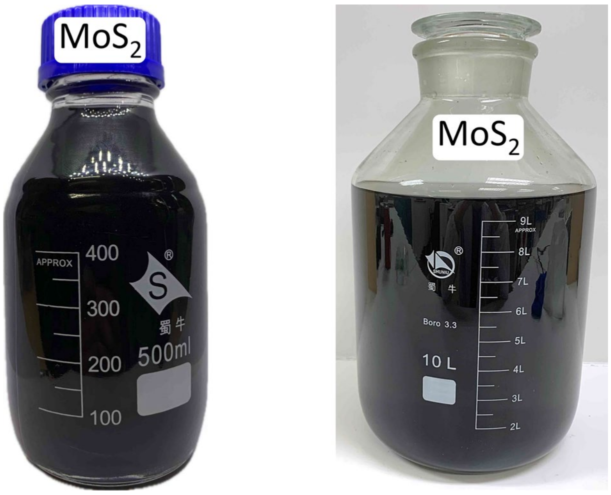

Figure S25. Scalable preparation of MoS_2 NSs.

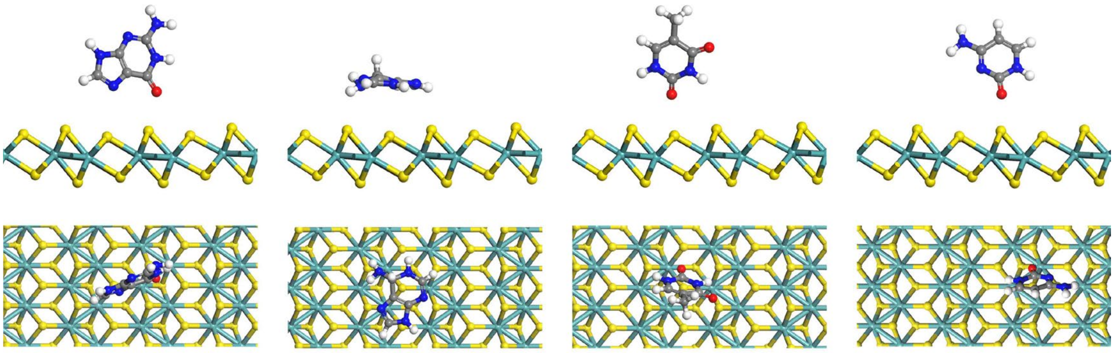

G A T C

Figure S26. The most stable binding configurations of G, A, T, and C bases on the 1T′-MoS_2.

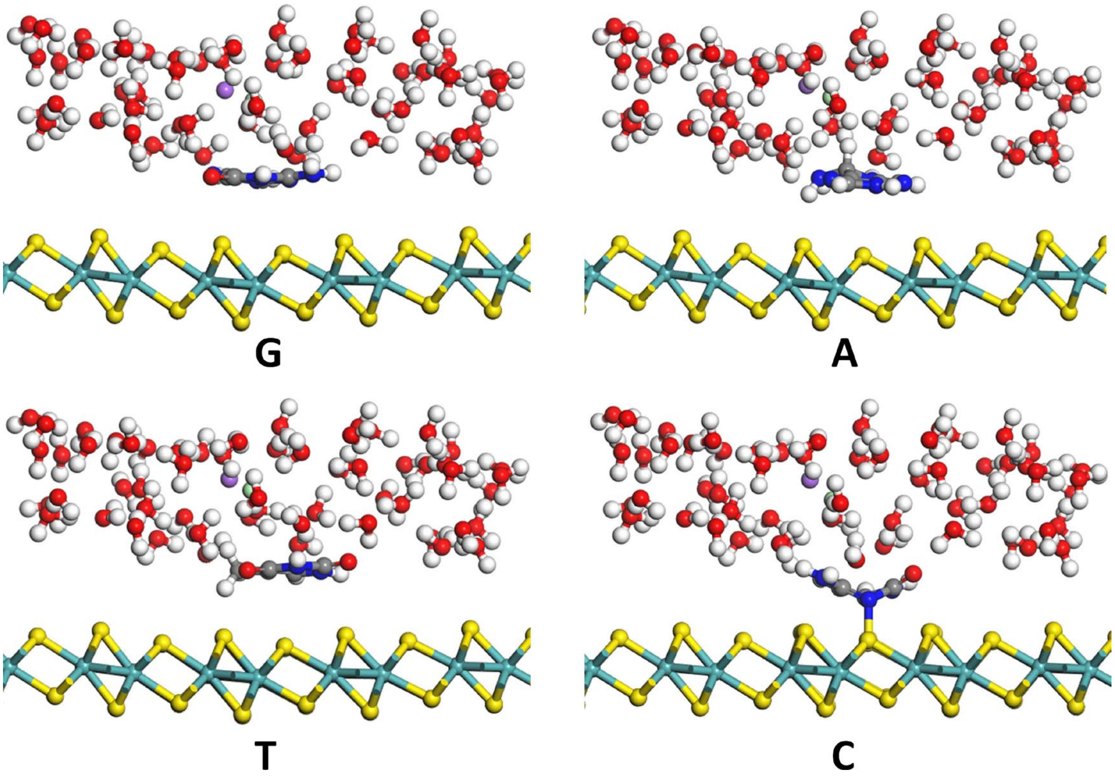

Figure S27. The adsorption configurations of DNA bases in parallel configurations after geometry optimizations with solvent environments. Blue balls = Mo, Yellow balls = S, Grey balls = C, Dark Blue balls = N, Red balls = O, and White balls = H.

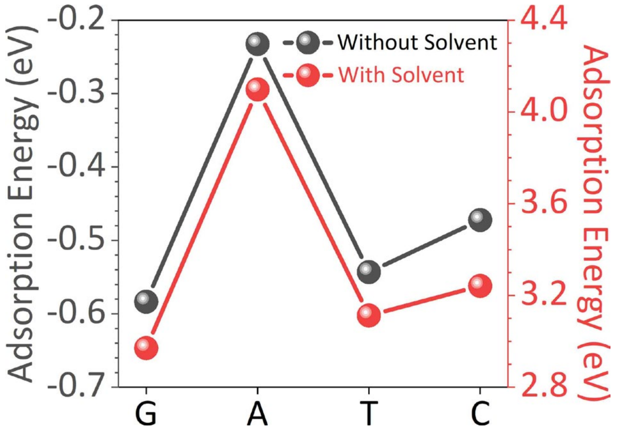

Figure S28. The adsorption energy comparisons of DNA bases for parallel configurations with/without solvent effect.

References

1. Mei L, Sun M, Yang R *et al*. Metallic 1T/1T′ phase TMD nanosheets with enhanced chemisorption sites for ultrahigh-efficiency lead removal. *Nat Commun* 2024; **15**: 7770.
2. Mei L, Gao Z, Yang R *et al.* Phase-switchable preparation of solution-processable WS_2 mono- or bilayers. *Nat Synth* 2025; **4**: 303-313.
3. Yang R, Mei L, Zhang Q *et al.* High-yield production of mono- or few-layer transition metal dichalcogenide nanosheets by an electrochemical lithium ion intercalation-based exfoliation method. *Nat Protoc* 2022; **17**: 358-377.
4. Mei L, Cao Z, Ying T *et al.* Simultaneous electrochemical exfoliation and covalent functionalization of MoS_2 membrane for ion sieving. *Adv Mater* 2022; **34**: 2201416.
5. Riaz T, Shehzad W, Viari A *et al.* ecoPrimers: inference of new DNA barcode markers from whole genome sequence analysis. *Nucleic Acids Res* 2011; **39**: e145-e145.
6. Martin M. Cutadapt removes adapter sequences from high-throughput sequencing reads. *EMBnet J* 2011; **17**: 10-12.
7. Bolyen E, Rideout J, Dillon M *et al.* Reproducible, interactive, scalable and extensible microbiome data science using QIIME 2. *Nat Biotechnol* 2019; **37**: 852-857.
8. How C, Ip J, Deconinck D *et al.* Refining sampling efforts for fish diversity assessment in subtropical urban estuarine and oceanic waters using environmental DNA with multiple primers. *Environ DNA* 2024; **6**: e70013.
9. R CORE TEAM, R. A language and environment for statistical computing. (2013).
10. Dixon P. VEGAN, a package of R functions for community ecology. *J Veg Sci* 2003; **14**: 927-930.
11. Clark S, Segall M, Pickard C *et al.* First principles methods using CASTEP. *Z Kristallogr Cryst Mater* 2005; **220**: 567-570.

12. Perdew J. Generalized gradient approximation made simple. *Phys Rev Lett* 1996; **77**: 3865-3868.

13. Hasnip P, Pickard C. Electronic energy minimisation with ultrasoft pseudopotentials. *Comput Phys Commun* 2006; **174**: 24-29.

14. Perdew J, Chevary J, Vosko S *et al.* Atoms, molecules, solids, and surfaces: Applications of the generalized gradient approximation for exchange and correlation. *Phys Rev B* 1992; **46**: 6671-6687.

15. Head J, Zerner M. A Broyden—Fletcher—Goldfarb—Shanno optimization procedure for molecular geometries. *Chem Phys Lett* 1985; **122**: 264-270.
